# Restoration of deuterium marker for multi-isotope mapping of cellular metabolic activity

**DOI:** 10.1038/s41598-025-33762-5

**Published:** 2026-01-07

**Authors:** Nadiia Yamborko, Laura Schwab, Lubos Polerecky, Yalda Davoudpour, Hugo Berthelot, Niculina Musat, Kim Milferstedt, Jérôme Hamelin, Hans-Hermann Richnow, Carsten Vogt, Hryhoriy Stryhanyuk

**Affiliations:** 1 Department of Technical Biogeochemistry, Helmholtz Centre for Environmental Research – UFZ, Permoserstraße 15, 04318 Leipzig, Germany; 2 Department of Soil Ecology, Helmholtz Centre for Environmental Research – UFZ, Theodor-Lieser-Straße 4, 06120 Halle, Germany; 3 Department of General and Soil Microbiology, Zabolotny Institute of Microbiology and Virology, NASU, Akademik Zabolotniy’s Str. 154, Kyiv, 03680 Ukraine; 4 Department of Earth Sciences, Faculty of Geosciences, Utrecht University, Utrecht, The Netherlands; 5 INRAE, University Montpellier, LBE, 102 Avenue Des Étangs, 11000 Narbonne, France; 6 Present Address: Institute of Biodiversity, Aquatic Geomicrobiology, Friedrich Schiller University Jena, Dornburgerstraße 159, 07743 Jena, Germany; 7 Present Address: Institute of Inorganic and Materials Chemistry, University of Cologne, Greinstraße 6, 50939 Cologne, Germany; 8 Present Address: Pelagos Laboratory, IFREMER, DYNECO, 29280 Plouzané, France; 9 Present Address: Department of Biology, Section for Microbiology, Aarhus University, Ny Munkegade 114, 8000 Aarhus C, Denmark; 10 Present Address: ISODETECT Leipzig, Deutscher Platz 5B, 04103 Leipzig, Germany

**Keywords:** Heavy water, Deuterium, Stable-isotope probing, Multi-isotope tracing, Metabolic activity, Oxygenic photogranule, Biochemistry, Biological techniques

## Abstract

Investigation of cellular metabolic activity with stable-isotope probing (SIP) implies the admittance of an isotope tracer into the metabolic pathway. Incubation with several isotope-markers (multi-isotope tracing) is required to trace nutrient metabolization and elucidate inter-cellular interactions in complex hosts and environmental communities. To cope with the lability of cell nutrition, deuterium in heavy ^2^H_2_^16^O water is employed as a substrate-independent general tracer of metabolic activity. However, the spatially-resolved deuterium tracing is hampered by detection limits due to its relatively low ionization yield and mass-interference issues. In the present work, we comprehensively assess the quantitation of deuterium incorporation into biomass employing the outstanding capabilities of nanoscale Secondary Ion Mass Spectrometry facilitating quantitative analysis of metabolic activity with single-cell or subcellular resolution. The effect of ion-probe-induced material relocation on the acquired pattern in ^2^H enrichment has been considered. Analytical expressions are suggested for the restoration of the deuterium fraction from the unresolved C_2_^2^H–C_2_^1^H_2_ mass-interference. Application of the suggested principle of equal relative assimilation and the multi-isotope tracing with the ^2^H-marker on a phototrophic symbiotic consortium paves the way to sensing the metabolic interplay among cells, recognition of homeostatic and shifted nutrition, checking for completeness of isotope-labelling and elucidation of nonlabelled substrate contribution.

**Supplementary Information:**

The online version contains supplementary material available at 10.1038/s41598-025-33762-5.

## Introduction

Information on microbial-mediated matter conversion scaled up to global biogeochemical cycles^1^ is required for the elaboration of resource-saving and energy-conversion approaches^2^. The combination of stable-isotope probing and nanoscale secondary ion mass spectrometry (SIP-nanoSIMS) has been efficiently employed to link metabolic activity with the identity of single-cells^3^ for tracing the intercellular nutrient flow and intracellular transformations e.g.^4–6^. In biological and biomedical research, stable-isotope tracers are introduced with isotope-labelled substances (e.g., ^15^N-thymidine, ^13^C-glucose, ^13^C-glutamine, ^13^CO_2_ gas, ^15^N_2_ gas, H^13^CO_3_^–^, ^15^NO_3_^–^, ^15^NH_4_^+^, ^2^H_2_O, H_2_^18^O) into the nutrition environment or growth medium.

Tracing the cellular activity in carbon, nitrogen and hydrogen assimilation at once (multi-isotope tracing) is required for a comprehensive investigation of substrate conversion and metabolic interactions in complex communities. Because of the irreversible ablation of material from a sample area explored upon a SIMS analysis, multi-isotope tracing requires simultaneous detection of secondary ions containing all tracer-isotopes, i.e., ^2^H^–^, ^13^C^–^ or ^13^C^14^N^–^ and ^12^C^15^N^–^.

For a proper decision on an optimal isotope-labelled substance, the lability of cellular nutrition (i.e., dependence of metabolic pathways and rates on nutrients’ chemical and isotopic composition) has to be considered to preserve intracellular homeostasis and to minimise the perturbation of native nutrition scenarios inherent to a studied ecosystem. Isotopic labelling of the growth medium with heavy water^7,8^ provides deuterium (^2^H) or heavy oxygen (^18^O) as a nutrient-independent tracer of cellular metabolic activity. Deuterated water (heavy ^2^H_2_^16^O water or the semi-heavy ^1^H^2^H^16^O) is a more preferred labelling source than heavy-oxygen water (^1^H_2_^18^O, ^1^H_2_^17^O or ^2^H_2_^18^O isotopologues) because the latter is much more expensive due to the more difficult separation of ^17^O and ^18^O containing isotopologues^9^.

The high relative mass difference between hydrogen isotopes leads to strong hydrogen isotope fractionation^10^ and may impede metabolism, i.e., cause toxicity effects already with 10% of ^2^H_2_^16^O fraction in the growth medium^11^. Incorporation of ^2^H from heavy water into biomass implies further dilution of the ^2^H tracer due to the major atomic fraction of hydrogen in biomass, e.g., 52 atomic percent (at%) according to the Redfield ratio for phytoplankton^12^.

In addition to biotic factors limiting the amount of the ^2^H tracer in analysed cells, the relatively low electron affinity of hydrogen atoms results in a moderate yield of secondary ^2^H^-^ ions when 16 keV Cs^+^ primary projectiles are employed for nanoSIMS analysis. Nevertheless, nanoSIMS has been successfully applied for ^2^H tracing in environmental studies^7,13^, in biology^14,15^ and in material science e.g.^16^. Strategies suggested for overcoming construction-related limitations of a serial NanoSIMS 50L instrument imply modification of factory-set hardware settings^13^, hardware upgrade and the derivation of ^2^H/^1^H isotope ratio from polyatomic ions^17^ along with the numerical restoration^7^ (see the Supplementary Information for more details, SI section S1).

The primary aim of the present work was to identify optimal isotopologue pairs within the [^2^H/^1^H, ^12^C^2^H/^12^C^1^H, ^16^O^2^H/^16^O^1^H, ^12^C_2_^2^H/^12^C_2_^1^H] series for quantitative high-resolution ^2^H mapping that could be done simultaneously with ^13^C and ^15^N tracing for studying the matter conversion and metabolic interaction in complex environmental systems. The demand for nanoscale lateral resolution is particularly strong when studying metabolic interactions in microbial consortia, where changes in structural composition occur at sub-micrometer spatial scales, even smaller than cell size. To demonstrate the advantages of the multi-isotope SIP-nanoSIMS technique, and particularly the prospects of ^2^H as a metabolic tracer in this type of applications, we used oxygenic photogranules (OPGs) as a model system. OPGs comprise a complex microbiome of syntrophically interacting heterotrophic and phototrophic bacteria^18^. This light-driven ecosystem is exchanging key metabolites when photosynthesis is active. OPGs have proven their effectiveness for wastewater treatment without external aeration^19^ and show potential to compete with the conventional activated sludge process^20^.

The approach of ^2^H mapping was optimized on resin-embedded maize-root samples and applied in multi-isotope tracing mode on OPGs. Due to the relatively high yield of C_2_^2^H^-^ ions and their robust biomass-featured spatial distribution, the C_2_^2^H/C_2_^1^H ratio was considered as a rather promising measure of the ^2^H fraction, despite the unresolved C_2_^2^H^-^ & C_2_^1^H_2_^-^ interference. The effect of this interference is reduced due to the decrease in C_2_^1^H_2_^-^ ion counts with increasing ^2^H fraction^14,21^. Together with the suggested new approach to restoring the ^2^H-fraction from polyatomic (C_2_^2^H^-^ & C_2_^1^H_2_^-^)/C_2_^1^H^-^ ion ratio, the employed principle of equal relative assimilation allowed the elucidation of interplay in nutrition channels and quantitative analysis of cellular metabolic interaction within the OPG consortium.

## Results and discussion

In the first stage of our study, the efficiency of deuterium mapping with ^2^H^-^, ^12^C^2^H^-^, ^16^O^2^H^-^ and ^12^C_2_^2^H^-^ ion species was evaluated on thick (~1 mm) resin-embedded maize root samples providing feasibility of long-lasting measurements necessary to reveal features of ^2^H spatial distribution in low-abundant ^2^H-containing ion counts and to clarify the mass-spectroscopy of corresponding molecular fragments. In the second stage, selected pairs of polyatomic ions were employed for multi-isotope tracing of metabolic activity in photo-hetero-trophic microbial associations (OPGs) enabling quantitative analysis of relative assimilation in carbon, nitrogen and hydrogen simultaneously. The details on multicollector settings, the restoration of ^2^H fraction from the unresolved ^12^C_2_^2^H & ^12^C_2_^1^H_2_ mass-peak, and the effect of an ion-probe-induced material relocation are explained in Supplementary Information (SI, sections S1-S3).

### Mass-spectroscopy of ^2^H-containing ion species

**Fig. 1:**
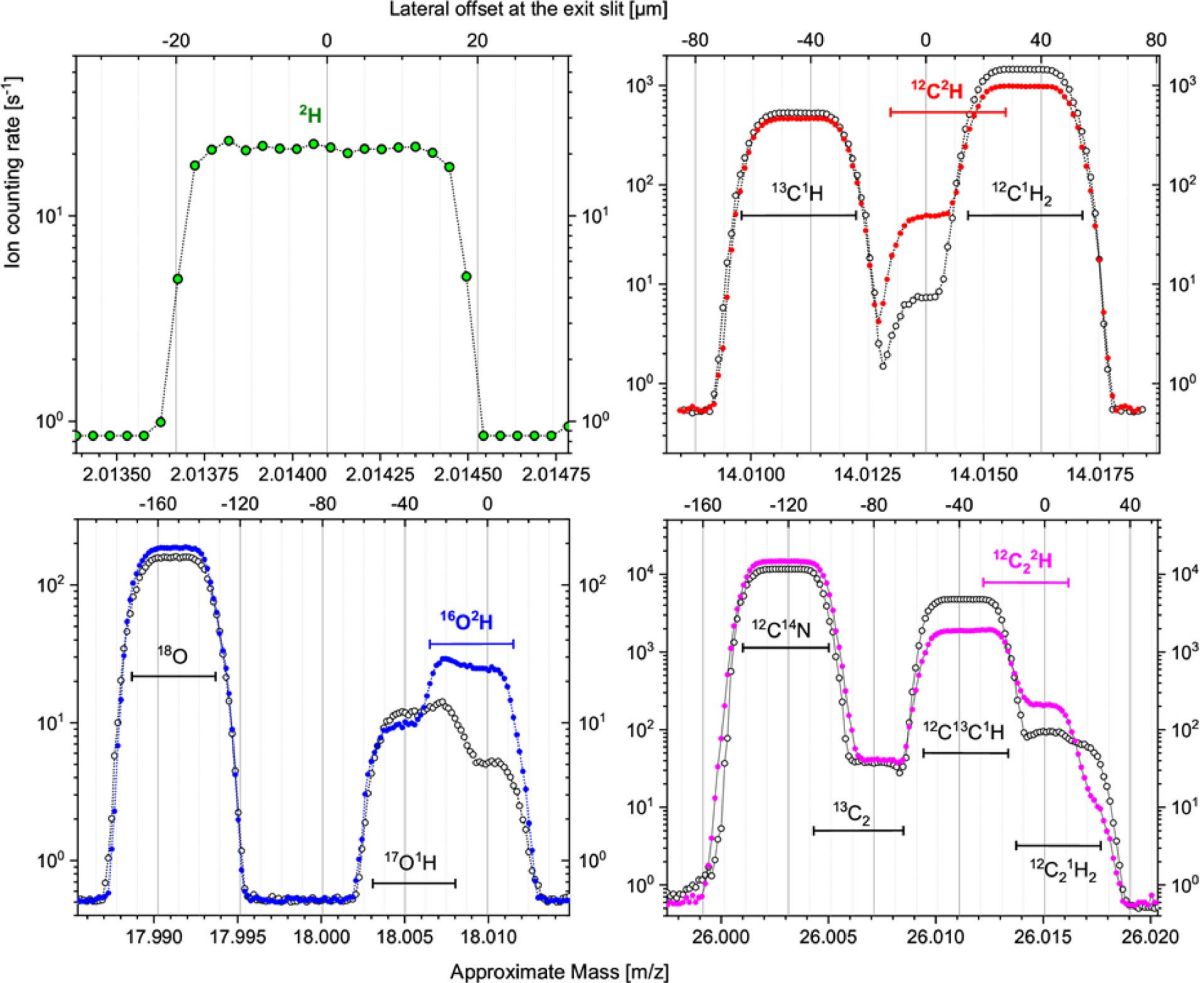
Mass-spectra acquired in the range of ^2^H, ^12^C^2^H, ^16^O^2^H and ^12^C_2_^2^H mass-peaks on ^2^H-labelled root sample (filled circles) and on a root of natural isotopic composition (open circles). Horizontal mass-range bars show neighbouring mass-peak positioning with 40 µm width of exit slits. Mass-peak centering is shown with the “0”-point in lateral upper x-axes. Spectra were averaged over 40 scans for 15×15 µm^2^ sample areas with 2.16 s accumulation per point in 0.15 V steps.

After carefull alignment of the secondary ion beam, the mass-resolving power (MRP) of about 16.000–18.000 was achieved in the mass-range of polyatomic ^2^H-containing ion species. Mass-spectra of ^2^H^–^, ^12^C^2^H^–^, ^16^O^2^H^–^ and ^12^C_2_^2^H^–^ ion-species were acquired from ^2^H-labelled and non-labelled maize-root samples (Fig. 1).

The mass-spectra were acquired with a defocused primary-ion beam in a 64×64 pixels raster over same-size areas of the ^2^H-labelled and non-labelled samples to keep counting rates of ^2^H-containing ion species for each sample comparable. Obtained counting rates are determined by the electron affinity of the corresponding molecular fragments and by their abundance within the analysed sample fragment. The mass-peak of ^2^H^–^ (Fig. 1, top left) reveals a count rate of 20 counts per second (cps) and is well separated from the mass-peak of ^1^H_2_^–^ for the ^2^H-labelled sample. The mass-peak of ^12^C^2^H^–^ with 50 cps is resolved from ^13^C^1^H^–^ (Fig. 1, top right) and appears as a clear flat-top shoulder at the low-mass side of a partly (≈50%) overlapping ^12^C^1^H_2_^–^ peak. The mass-peak of ^16^O^2^H^–^ (Fig. 1, bottom left) delivers 25 cps and overlaps partly (≈50%) with the ^17^O^1^H^–^ peak, but it can still be employed for mass-spectrometric quantitation of ^2^H content when ^16^O^1^H_2_^–^ ion species are not revealed at the high-mass side. Regarding the mass-peak of ^12^C_2_^2^H^–^, a relatively high counting rate of 200 cps is delivered from the ^2^H-labelled sample (Fig. 1, bottom right). However, it overlaps to 40% with the mass-peak of ^12^C^13^C^1^H^–^and over 80% with that of ^12^C_2_^1^H_2_^–^, where the latter decreases in count rate from 65 cps down to 10 cps when the non-labelled (natural ^2^H abundance) and ^2^H-labelled samples are compared. This feature of the ^12^C_2_^1^H_2_^–^ dependence on the ^2^H-label content has previously been emphasized ^21^ and is comprehensively considered in the present study for the restoration of the ^2^H fraction from the unresolved ^12^C_2_^2^H & ^12^C_2_^1^H_2_ mass-peak.

### Direct measurements and the restoration of natural ^2^H abundance

**Fig. 2:**
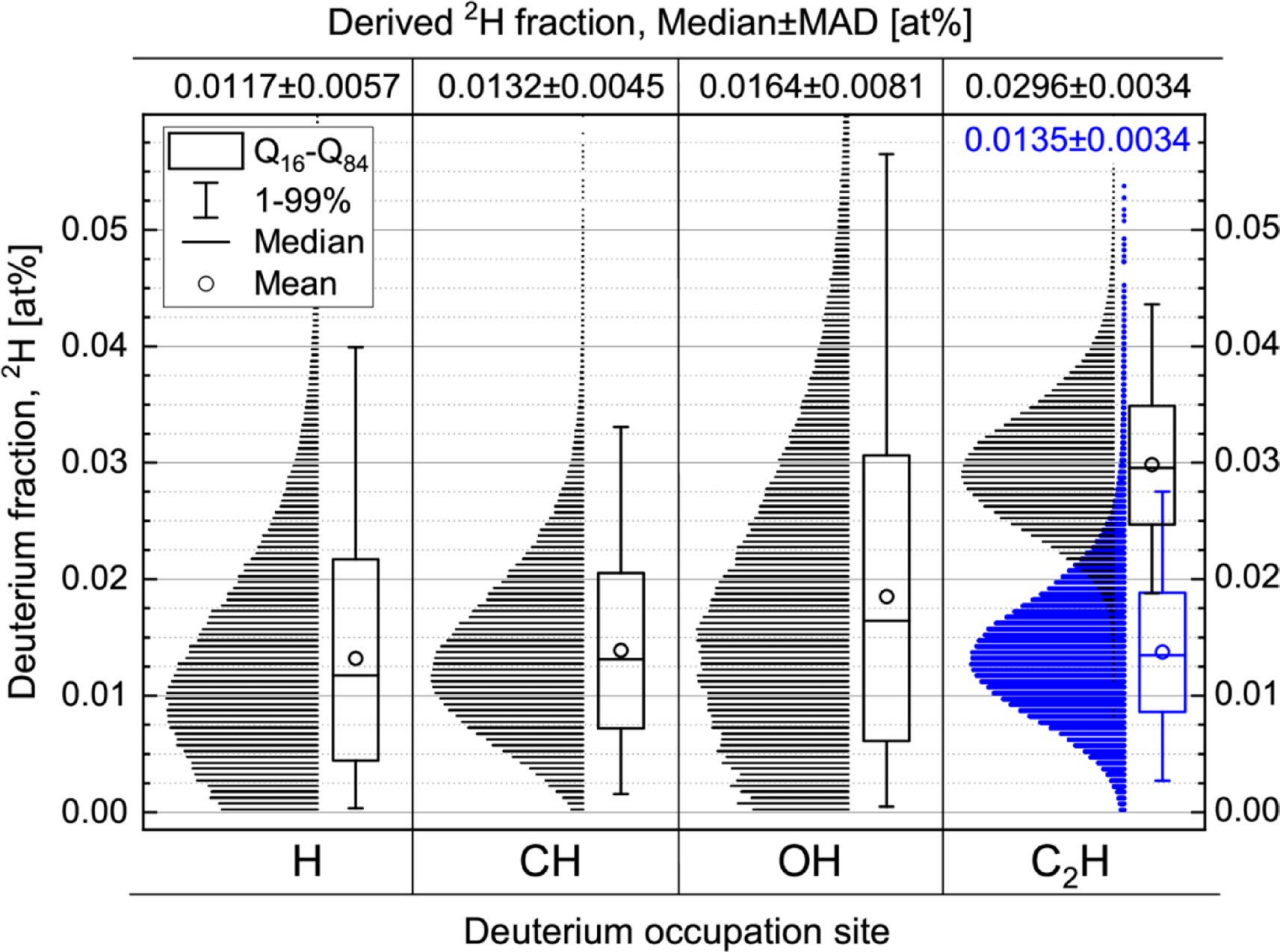
Single-pixel distribution in ^2^H fraction derived from the hydrogen isotopic ratio in H, CH, OH and C_2_H isotopologue pairs acquired from a resin-embedded maize-root sample with a natural isotopic composition. The distribution, restored from the unresolved ^12^C_2_^2^H & ^12^C_2_^1^H_2_ peak according to Eq. (1) is shown in blue.

The fraction of ^2^H in a resin-embedded maize-root sample with a natural isotopic composition was analyzed in the imaging mode of nanoSIMS 50L employing multicollector settings II & III (see Table S1 in SI). The distributions of the measured values among the image pixels are shown with histograms and box-plots in Fig. 2 separately for ^2^H fractions derived from the H, CH, OH and C_2_H isotopologue ion count ratios.

The median value of the ^2^H fraction is close to the natural ^2^H abundance (fraction) ${F}_{0}$=^2^H/(^2^H+^1^H)×100=0.0115 at% when derived from H, CH and OH isotopologue ratios, but appears to be about 2.5 times overestimated (${F}_{0}^{\prime}$=C_2_^2^H/(C_2_^2^H+C_2_^1^H)×100=0.0296 at%, see Fig. 2) due to the unresolved ^12^C_2_^2^H & ^12^C_2_^1^H_2_ mass peaks (see Fig. 1 and S1). Nevertheless, the high counting rate of ^12^C_2_^2^H^−^ ion species offers an enhanced precision in the quantitation of ^2^H fraction. Moreover, the ion-beam induced material-smearing effect, revealed strongly in ^2^H maps derived from H, CH and OH isotopic ratios (Fig. S4), is almost not detectable when C_2_H isotopologues are considered for the restoration of the ^2^H fraction (Fig. S5 d). Thus, despite the unresolved ^12^C_2_^2^H & ^12^C_2_^1^H_2_ mass interference, one still has two solid advantages (i.e., enhanced precision and negligible smearing effect) of C_2_H isotopologue ratio consideration for ^2^H mapping. With this motivation, a new method for the restoration of ^2^H fraction with the unresolved ^12^C_2_^2^H & ^12^C_2_^1^H_2_ mass peaks has been developed in the present work (SI, section S2).

The suggested restoration of ^2^H fraction can be implemented with a rather simple expression. In terms of isotope ratio, the restored ^2^H/^1^H value is expressed as

1 $$R_{r} = R^{\prime } - \varepsilon_{{H_{2} /H}} ,$$

where ${R}^{\prime}$ is the hydrogen isotope ratio overestimated due to the unresolved ^12^C_2_^2^H, and the correcting parameter ${\epsilon }_{{H}_{2}/H}$ is defined as the C_2_H_2_^−^/C_2_H^−^ ion-yield ratio (section S2 in SI). The calculation of ${\epsilon }_{{H}_{2}/H}$ value employs the difference between CH and C_2_H isotopologue ratios (${R}_{0}$ and ${R}_{0}^{\prime }$, respectively) acquired on the unlabelled sample with a natural ^2^H abundance (see S2 section in SI for more details).

2 $$\varepsilon_{{H_{2} /H}} = \left( {R_{0}^{\prime } - R_{0} } \right) \times (1 + R_{0} ) = { }\left( {1.6411 \pm 0.0012} \right) \times 10^{-4}$$

The median value of the ^2^H fraction restored from the unresolved ^12^C_2_^2^H & ^12^C_2_^1^H_2_ peak employing the Eq. (1) (shown in blue in Fig. 2) is close to the value of the ^2^H abundance ${F}_{0}$=0.0132±0.0045 at% derived from the CH isotopologue ratio ${R}_{0}$ accepted in Eq. (2) for the calculation of ${\epsilon }_{{H}_{2}/H}$ value.

The distributions derived from the H and OH isotopologue ratios are more stretched and even truncated considerably at 0 at% due to the relatively low counting rates for ^2^H^–^ and ^16^O^2^H^–^ ions (see Fig. 1). In case of such truncated distributions, it may be better to consider a modus value instead of the median. The most stretched distribution, as observed for the OH isotopologues, may be due to the contribution of ^16^O^1^H_2_^–^ ion species that are not revealed in the mass-scan at 18 amu for the studied resin-embedded samples (Fig. 1) but may show a higher yield from other sample types.

### Application on a complex microbial community

With the established multi-isotope tracing approach, the SIP-nanoSIMS methodology was applied on oxygenic photogranules (OPG) to check its applicability and prospects for studying metabolic interactions in the complex microbial consortia. OPGs are microbial aggregates with diameters ranging from several hundreds of micrometers to four to five millimeters. These roughly spherical aggregates float freely in an agitated aqueous system, but settle rapidly when mixing is stopped. OPGs harbor a microbial community dominated by heterotrophic and phototrophic bacteria. These two groups of microorganisms are believed to syntrophically exchange heterotrophically produced CO_2_ and photosynthetically produced O_2_^18^. CO_2_ may be produced from an externally provided carbon source, for example, organic compounds contained in wastewater. Alternatively, hetetrotrophs may produce CO_2_ from the conversion of mainly phototrophically produced extracellular polymeric substances contained in the photogranules. This is the case in our experiment where only inorganic carbon is added in form of HCO_3_^-^ as it can only be assimilated by autotrophic microorganisms. In studied photogranules, the majority of autotrophs are phototrophic filamentous cyanobacteria of the order Oscillatoriales^18^. These cyanobacteria are not known to fix N_2_ in the presence of another more accessible nitrogen source. The ^15^N-labelled ammonium chloride was provided as a nitrogen source for heterotrophs and phototrophs. Deuterated water was providing ^2^H as a tracer of biosynthetic OPG activity.

With the reliable ^2^H reconstruction from the unresolved ^12^C_2_^2^H & ^12^C_2_^1^H_2_ mass-peak (described in SI, section S2), the correcting parameter ${\epsilon }_{{H}_{2}/H}$=(1.4750±0.0014)×10^−4^ was derived and the multicollector settings V (Table S1) were adopted for simultaneous tracing of H, C, N isotopes in parallel with the mapping of cellular ^32^S and ^31^P (^31^P^1^H, see Fig. S6) upon the analysis of OPG samples.

**Fig. 3:**
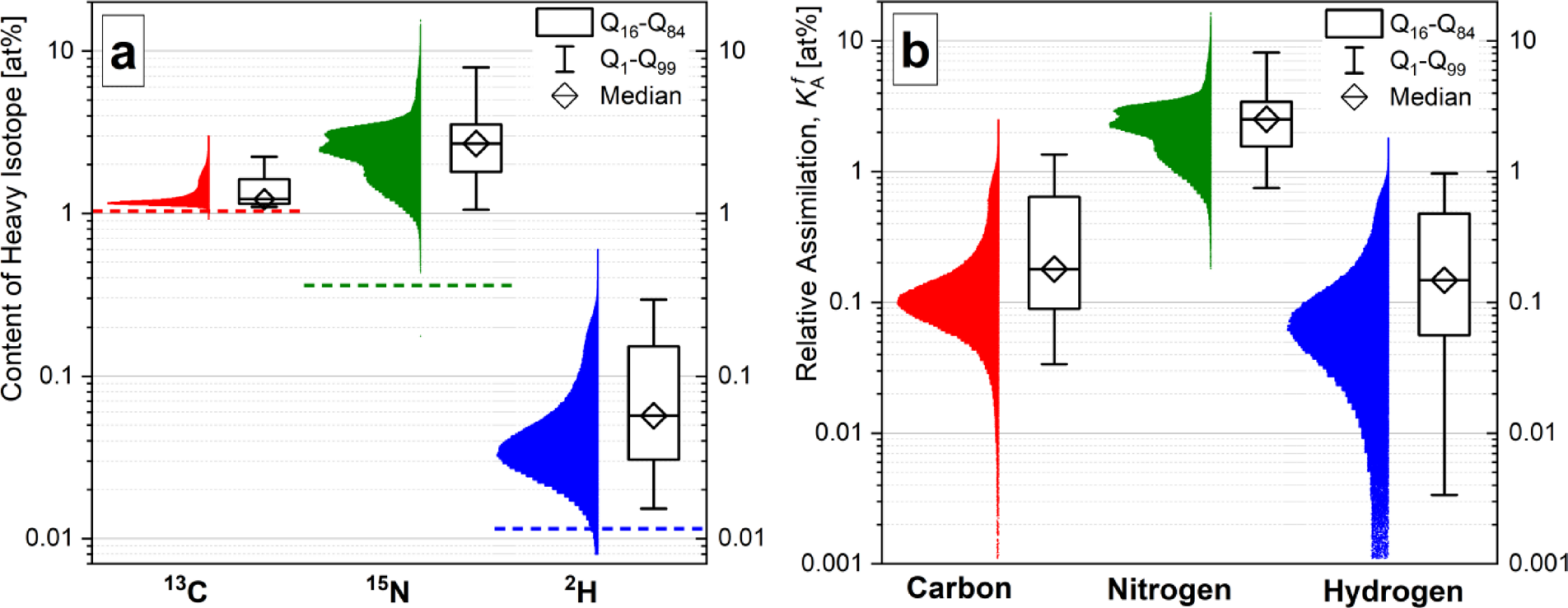
Distribution histograms of biomass-related pixels in the ^2^H, ^13^C and ^15^N fractions (**a**) and in the relative H, C and N assimilation (**b**) for 5 analyzed areas of OPG thin section. Horizontal dashed lines in the frame (**a**) show the natural abundance of the corresponding isotopes. The boxplots next to the histograms summarize the distributions with their median and the Q_16_ and Q_84_ quantiles. Interquantile Q_1-99_ range is shown with horizontal whiskers.

The histograms of biomass-related pixel distribution according to the biomass isotopic enrichments in ^2^H, ^13^C and ^15^N (Fig. 3a) are not directly comparable because of the different natural abundance of these isotopes (shown with horizontal dashed lines) and due to their different fractions in the growth substrate. Isotopic enrichment of each biomass volume-unit (voxel of about 60×60×100 nm^3^, 3D analog of a 2D-pixel) within cell-confining regions of interest (RoIs, see RoI-definition example in Fig. S7) was derived from corresponding ion-count ratios and converted with the Eq. (3) into the relative assimilation ${K}_{A}^{f}$ (Fig. 3b) representing the assimilated amount ${E}_{a}$ of tracer-derived H, C or N expressed relatively to the final cellular amount ${E}_{f}$ of the corresponding element achieved after the incubation^22^.

3 $$K_{A}^{f} = \frac{{E_{a} }}{{E_{f} }} = \frac{{R_{f} - R_{i} }}{{\left( {1 + R_{f} } \right) \times \left\{ {F_{gs} \times \left( {1 + R_{i} } \right) - R_{i} } \right\}}} = \frac{{F_{f} - F_{i} }}{{F_{gs} - F_{i} }}$$

In the Eq. (3), $R$ is the heavy-to-light isotope ratio, $F$ is the corresponding atom fraction of heavy isotope, $F=R/(R+1)$, subscripts *i* and *f* refer to the values before and after the incubation (*initial* and *final*), and ${F}_{gs}$ is the fraction of heavy isotope in the isotope-labelled tracer-substance contributing to the growth substrate.

The relative assimilation has also been calculated for each single cell as the mean value over biomass volume-units (voxels) within the corresponding cell-confining RoI (shown with yellow contours in Fig. S7a,b). Fig. S9 shows the distribution of single-cells in their relative assimilation of carbon, nitrogen and hydrogen to be well reproduced with the distribution of biomass-units in the corresponding relative assimilation calculated for each cellular-related voxel. Features of the cell distribution in relative assimilation are better revealed with a higher count of subcellular biomass volume-units (voxels). Therefore, voxel-resolved data were considered for quantitative evaluation of the relative elemental assimilation in the present work.

To account for the dilution of ^13^C and ^2^H isotope-tracers with fixative^23^ and embedding materials, values of relative assimilation $K_{A}^{f}$(^12^C^13^C/^12^C_2_) and $K_{A}^{f}$(^12^C_2_^2^H/^12^C_2_^1^H), computed with the corresponding C_2_ and C_2_H isotopologue ratios, were corrected with 1.136 multiplication factor and 0.03 at% offset, derived as the slope *b* and y-intercept *a* of the linear function fitting ${K}_{A}^{f}\left({}_{ }{}^{13}\text{C}{}_{ }{}^{14}\text{N}/{}_{ }{}^{12}\text{C}{}_{ }{}^{14}\text{N}\right)=a+b\times {K}_{A}^{f}\left({}_{ }{}^{12}\text{C}{}_{ }{}^{13}\text{C}/{}_{ }{}^{12}{\text{C}}_{2}\right)$ relation (see Fig. S10).

**Fig. 4:**
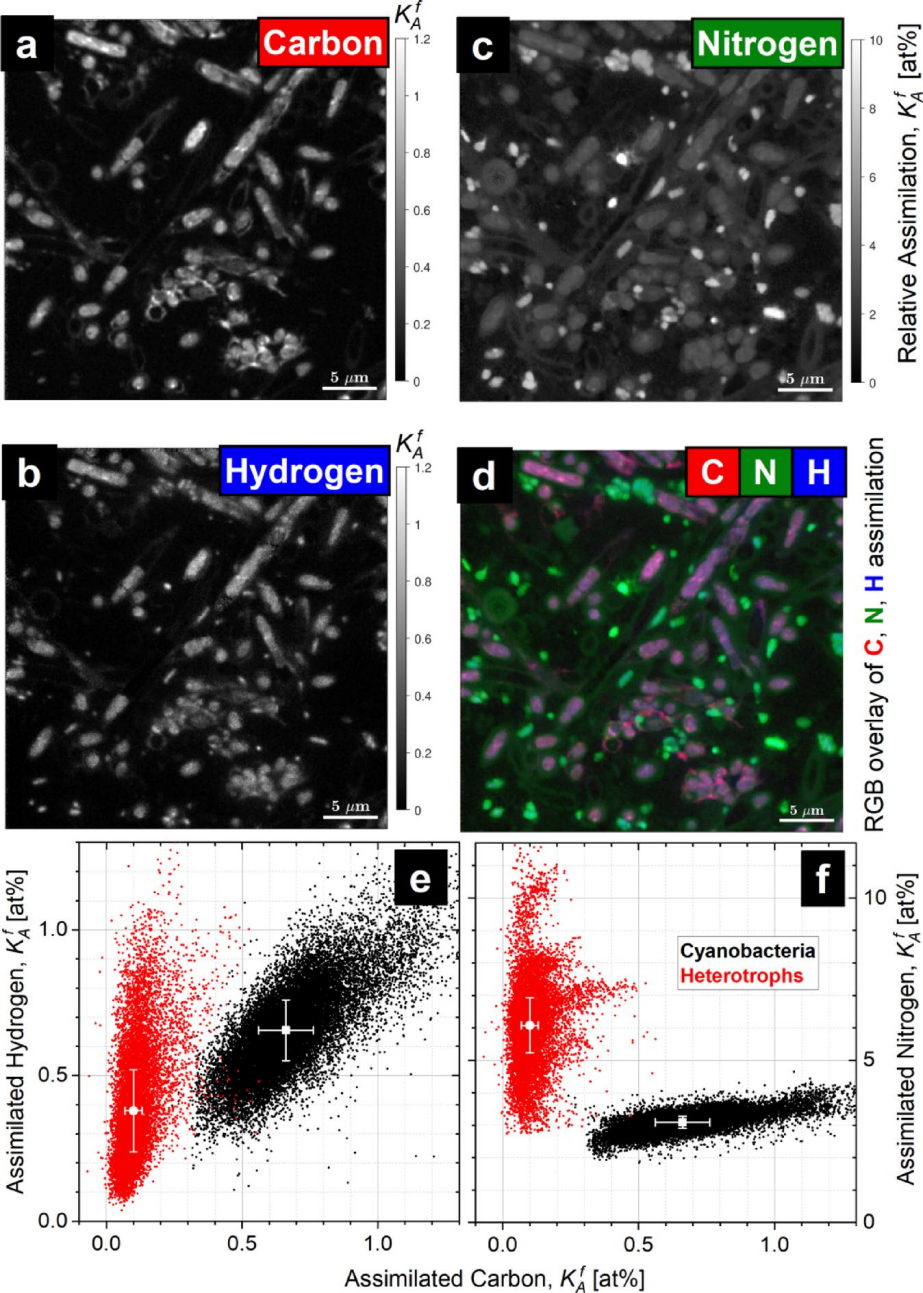
Relative metabolic activity ${K}_{A}^{f}$ of complex microbial consortium in oxygenic photogranules (OPG) revealed with multi-isotope (^2^H, ^13^C, ^15^N) SIP-nanoSIMS. Separate frames show the relative assimilation in fraction (at%) of assimilated elements: carbon – frame a; hydrogen – frame b; nitrogen –frame c. Frame d shows the RGB-overlay of frames a, b and c. Frames e, f: scatterplots of pixels assigned to the biomass of cyanobacteria (in black) and to heterotrophs (in red) according to the fraction of assimilated carbon and hydrogen – frame e; and according to carbon and nitrogen relative assimilation – frame f; median values and median absolute deviations (Med±MAD) are shown with white symbols.

In Fig. 4, the spatial distribution of cellular relative assimilation is visualized in a thin section through the outer green part of a photogranule. Distribution of single-cells in relative assimilation activity ${K}_{A}^{f}$ (Fig. 4a-d) facilitates the differentiation between consortium-representative species via quantitative analysis of their elemental assimilation efficiency. With the map derived for the relative carbon assimilation in the OPG-consortium (Fig. 4a) its autotrophic members fixing ^13^CO_2_ can be clearly recognized as elongated cells arranged in filaments (see Fig. S7 a,c) typical for cyanobacteria usually found in OPG ^18^. Complete filaments are not seen in the figures, as the imaged area represents a thin section on which only part of the filaments happens to be positioned in the sectioning plane. All metabolically active cells in OPG show the fraction of hydrogen incorporated from water (Fig. 4b) to be in a similar range as the fraction of carbon supplied via ^13^CO_2_ fixation by autotrophs (Fig. 4a,e, y-axis).

One can recognize cyanobacteria cells (yellow RoIs in Fig. S7a) with almost equal relative assimilation of C and H (Fig. 4e, black). This feature in the metabolic activity of cyanobacteria would suggest their homeostatic nutrition keeping cellular elemental Redfield ratio almost constant, however the availability of ^15^NH_4_^+^ boosts synthesis of N-rich molecules (e.g., proteins) revealed as about fivefold increase in relative assimilation of N (Fig. 4f, black). Cyanobacteria are known to store nitrogen in the form of so-called structured granules enriched in cyanophycin granule polypeptide^24,25^. Nevertheless, proportional assimilation of carbon and nitrogen makes it easy to recognize cyanobacteria when overlaying frames a, b and c of Fig. 4, yielding Fig. 4d.

Cyanobacteria are coloured in the RGB-overlay from bright pink (slightly prevailing C assimilation over H assimilation) to magenta and purple spots with ^2^H prevailing assimilation. Bright pink-red areas can be identified also in between cyanobacterial cells (Fig. 4d, lower part) and may be ascribed to carbon-rich extracellular polymeric substances (EPS) synthesised de novo from photosynthetically assimilated ^13^CO_2_. Typical cyanobacteria found in OPG belong to the order *Oscillatoriales*, which are filamentous gliding cyanobacteria^18^. Bright EPS spots immediately adjacent to cyanobacterial cells could represent EPS excreted for the gliding motility.

All cells appearing bright green in the overlay (Fig. 4d) may be assigned to heterotrophic bacteria (marked with yellow RoIs in Fig. S7b) possessing intensive nitrogen assimilation (Fig. 4c,d in red) from the supplied ^15^NH_4_^+^. This high nitrogen assimilation (Fig. 4f in red, y-axis; Table 1, column III) implies the obvious necessity of carbon recycling from the unlabelled carbon sources, e.g., EPS or dissolved organic matter pool (DOM), for maintaining the elemental biomass-stoichiometry upon the limited carbon supply with ^13^C-labelled phototrophic exometabolites from cyanobacteria (Fig. 4e,f in red, x-axis; Table 1, column I). These heterotrophic bacteria consume ^2^H from water for fatty-acid synthesis involving NADPH/NADP^+^, whereas more ammonium-derived ^1^H is supplied together with ^15^N for protein synthesis resulting in slightly lower ^2^H enrichment of heterotrophs as compared with cyanobacteria (Fig. 4e, y-axis; Table 1, column V).

**Table 1:**
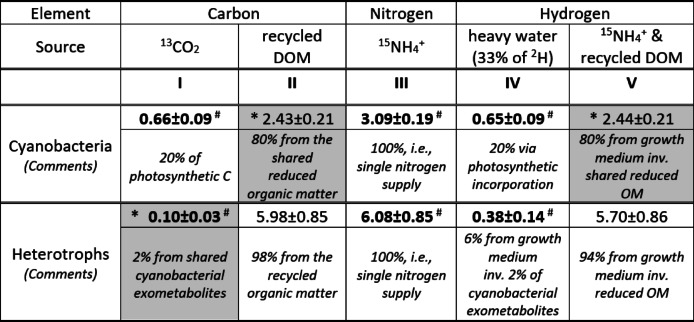
Element- and source-resolved distribution of the relative assimilation ${K}_{A}^{f}$ represented for OPG members with median values and median absolute deviation as Med±MAD [at%].

Values derived from the SIP-nanoSIMS data ^#^are shown in bold (with white symbols in Fig. 4e,f). Assimilation due to the syntrophic interaction between cyanobacteria and heterotrophs is shown in *grey-filled cells.

With 1 mM ^15^NH_4_^+^ as a not-limiting nitrogen source in the nutrition medium, its relative assimilation reached 10% for heterotrophic cells (Fig. 4c,f in red), whereas 2–3 times lower N-assimilation activity of cyanobacterial filaments (Fig. 4f in black) was revealed due to their rate-limiting carbon supply via CO_2_ fixation. It is important to note the distribution pattern of bacterial cells around phototrophs – they are always close neighbours with a mutualistic interaction between phylogenetically unrelated members in the OPG community.

Assuming the elemental stoichiometry of each OPG member remains stable^26^ during the time of incubation with an isotope-labelled substrate, one may expect close median values of relative assimilation (${K}_{A}^{f}$) in all elements (H, C and N) for representatives within each group (autotrophs and heterotrophs). With this assumption and the median values of relative assimilation derived with the isotope-labelled nutrition part (Fig. 4e,f; median values shown with white symbols), the fractions of carbon and hydrogen from the recycled DOM and ^15^NH_4_^+^-related hydrogen, assimilated by cyanobacteria and heterotrophs Table 1 (columns II and V), were derived as follows.

With its non-limiting content in the growth medium, ^15^NH_4_^+^ was considered as the easily-accessible prevailed nitrogen source providing all, i.e., 100% of cellular nitrogen gained by OPG consortium during the incubation. The relative nitrogen assimilation of 3.09±0.19 at% (${K}_{A}^{f}$, Med±MAD) for cyanobacteria and 6.08±0.85 at% for heterotrophs (Table 1, column III; Fig. 4f, y-axis, median values shown with white symbols) imply twofold difference in their nitrogen-specific growth rate

4 $$\gamma = \frac{{ - \log_{2} \left( {1 - K_{A}^{f} } \right)}}{t} = - \frac{{\ln \left( {1 - K_{A}^{f} } \right)}}{t \times \ln \left( 2 \right)}$$

5 $$\Delta \gamma = \frac{\partial \gamma }{{\partial K_{A}^{f} }} \times \Delta K_{A}^{f} = \frac{1}{t \times \ln \left( 2 \right)} \times \frac{1}{{\left( {1 - K_{A}^{f} } \right)}} \times \Delta K_{A}^{f}$$

returning 0.76±0.05 at%/h and 1.51±0.22 at%/h respectively with the cultivation time $t$=6 h.

Carbon fractions supplied from ^13^CO_2_ via photosynthesis (0.66±0.09 at%) and exometabolite sharing (0.10±0.03 at%, syntrophic share with heterotrophs) by cyanobacteria were derived from nanoSIMS data as ${K}_{A}^{f}$ median values (Table 1, column I) shown in Fig. 4e,f (x-axes, median-value symbols in white). Carbon fraction assimilated from the recycled DOM (Table 1, column II) by cyanobacteria (2.43±0.21 at%, share from heterotrophic OM reducers) and by heterotrophs (5.98±0.85 at%) was calculated as the difference between the corresponding fraction of assimilated nitrogen (column III) and the fraction of carbon from ^13^CO_2_ (column I). Such a calculation is valid when i) the ^15^NH_4_^+^ can be considered as the prevailed nitrogen source and ii) the assumed above preservation of biomass stoichiometry is fulfilled implying equal relative assimilation of all elements (C, N and H) by each group of OPG consortium. The cellular fraction of hydrogen originating from the supplied ^15^NH_4_^+^ and recycled DOM (column V) was calculated for cyanobacteria (2.44±0.21 at%, involving a major share from heterotrophic OM reducers) and for heterotrophs (5.70±0.86 at%) as the difference between the fraction of assimilated nitrogen (column III) and the hydrogen fraction incorporated from heavy water (Table 1, column IV) shown in Fig. 4e (y-axis, median-value symbols in white).

For a microbial group with the elemental stoichiometry of its biomass preserved, one may estimate the contribution of non-labelled nutrition sources with either i) an exclusive source of at least one element (C, N or H) isotope-labelled, or with ii) a known value of the growth- or division-rate for a target cell-type of a studies consortium. Assuming the DOM to be depleted in nitrogen, we considered ^15^NH_4_^+^ as an exclusive nitrogen source for OPGs in this study. This assumption allowed for the quantitative evaluation of non-labelled nutrient supply (Table 1) and for the calculation of cell-division rates (Eqs. (4) and (5)) for cyanobacteria and heterotrophs. In another way, relative assimilation ${K}_{A}^{f}$ may also be calculated with a known growth-rate value *µ* for the biomass of a target cell-type or corresponding cell-division rate γ.

6 $$K_{A}^{f} = 1 - e^{ - \mu \times t} = 1 - 2^{ - \gamma \times t}$$

With the value of relative assimilation acquired in this way, one can further analyse the completeness in isotope-labelling of nutrient supply by comparing the Eq. (6) output with ${K}_{A}^{f}$ values derived from the changes in isotopic composition of a corresponding cell-type upon their incubation with an isotope-labelled substrate.

The photogranules that were incubated in the experiments here had a net heterotrophic activity. The required carbon for this activity was derived from the supplied ^13^CO_2_ and via EPS conversion. EPS is typically present in sample quantities in photogranules as it is produced for example for cyanobacterial gliding motility. However, contrasting environmental conditions, e.g., illumination, as well as photogranule properties, e.g., size, may partition autotrophic and heterotrophic activities differently. Especially for biotechnological applications of photogranule biomass, controlling the specific activities of the photogranule community by varying the environmental conditions are of interest. The procedure of metabolic flux analysis suggested here allows us to generate these data in further experiments. Our study of metabolomic exchange between cyanobacteria and other bacteria in photogranules shows the expected metabolic shifts in carbon fixation (Calvin cycle pathway) depending on light availability^27^.

It is worth noting that the fraction of hydrogen incorporated from heavy water within 0.001–1 at% range (Fig. 3b) was analysed with NanoSIMS 50L delivering good ion-counting statistics from each biomass unit confined in single voxels (3D-pixels). The dynamic range of hydrogen isotopic analysis starting from its natural abundance (Figs. 2 and 3a) facilitates the efficient differentiation between single-cells and cellular compartments according to their metabolic activity. The incorporation of hydrogen from heavy water (Fig. 4e, y-axis) is revealed for all types of cells regardless of functional activity and nutrition mode (whether autotrophic or heterotrophic). In this way, ^2^H from water is proved as a universal tracer in all biosynthetic processes being primarily supplied to the sites of the most intensive biosynthesis like DNA/RNA-synthesis sites and synthesis of service enzymes around bacterial chromosome (see the overlay of P and H-incorporation maps in Fig. S8). This mode of stable-isotope labelling (^2^H from deuterated water) allows the tracing of dividing cells in a population^14^. Hydrogen is incorporated from water into cell metabolism by either hydrolysis, osmotic interactions, or most importantly by hydrogen incorporation into NADPH synthesis^28^. NADPH is a co-factor for metabolic reactions in the Calvin cycle, lipid and nucleic acid syntheses, where NADPH is required as a reducing agent (hydrogen source). NADPH and its oxidized form NADP^+^ are utilised by all forms of cellular life^29^.

## Conclusions and outlook

The multi-isotope tracing of metabolic activity in a complex OPG microbiome allowed us to differentiate between two populations of phototrophic and heterotrophic bacteria and to reveal the syntrophic interaction between these inhabitants of the phototrophic symbiotic consortium via analysis of the interplay in their nutrition channels. With the example of nutrition analysis on the OPG consortium, we demonstrated the prospects in application of the suggested approach for e.g., optimization of wastewater treatment processes. The employed stoichiometry-preservation principle constitutes a framework for metabolic flux analysis in complex systems, implying each representative group of the studied consortium shows equal relative assimilation of different elements (e.g., C, N and H).

Homeostatic ^13^CO_2_ fixation was revealed for autotrophic inhabitants of OPG consortium with relative assimilation of carbon following the fraction of hydrogen incorporated from heavy water (Figs. 4e, S9 a, black). In this case, the complete isotope-labelling of phototrophic nutrition channel was achieved with heavy ^2^H_2_O water and ^13^CO_2_ involved in the photosynthetic assimilation process. Major fraction (80–98%) of assimilated carbon and hydrogen was found to be supplied via recycling of OM (Table 1, columns II, V) converted by heterotrophic reducers to DOM, i.e., beyond the isotope-traced pathway. Such an incomplete isotope-labelling of nutrient supply is revealed as a considerable discrepancy in the relative assimilation of different elements within a defined geno- or phenotypic group of a microbial consortium^30^.

The study results emphasised the integrating role of ^2^H labelling through deuterated water in complex systems with various life strategies and metabolic types. All metabolically active cells in the studied OPG consortium revealed the incorporation of deuterium from heavy water. Unlike ^13^C and ^15^N tracers, a major fraction of ^2^H-marker from heavy water stays incorporated into the biomass upon cellular metabolic activity (i.e., detectable with nanoSIMS). For example, the respiration process implies the synthesis of NADH, NADPH etc. with incorporation of ^2^H from heavy water, whereas ^13^CO_2_ is released.

The achieved reliable restoration of deuterium fraction already from its natural 0.0115 at% abundance proves SIP-nanoSIMS capability to sense and localize extremely low biosynthetic activity with relative assimilation ${K}_{A}^{f}$ starting in the~0.001 at% range (Fig. 3b) corresponding to metabolic rate of~10^–5^ h^-1^ upon cellular maintenance^31^. Employing this high sensitivity for the quantitation of metabolic heterogeneity among single cells of a microbial population^32^ makes it feasible to trace microevolutionary processes and reveal obvious and non-obvious anthropogenically elicited changes in the environment.

## Methods

### Multi-isotope labelling and preparation of oxygenic photogranules

OPGs were grown in a sequential batch reactor as previously described^33^. The photogranules were transported in darkness at room temperature to the nanoSIMS facility at UFZ in Leipzig. Isotope labelling incubations were performed under light in a medium containing heavy water (33 vol.% of ^2^H_2_O), H^13^CO_3_^-^ (^13^CO_2_ – 87 at% ^13^C) and ^15^NH_4_^+^ (93 at% ^15^N). The final concentrations of the labelled nutrients were 1 mM ^15^NH_4_^+^ and 2 mM H^13^CO_3_^-^. After 6 h of incubation, OPGs were fixed with 2% paraformaldehyde in cacodylate buffer (pH 7.4, 0.1 M) at 4˚C overnight and washed two times with the buffer. Dehydration was performed with ethanol series (30%, 50%, 70%, 80%, 90%, and 3 times 100%) for 15 min each. Low-viscosity LR White resin infiltration was done using 1:3, 1:2, 1:1, 2:1 and 3:1 of resin:ethanol mixture, each for 45 min, followed by pure LR White resin for one hour and overnight. Finally, the resin-infiltrated OPG sample was cured in an oven at 60 °C for two days. The polymerized sample block was trimmed using a Leica EM TRIM2 trimmer and sectioned with Leica EM UC7 ultramicrotome employing a freshly-prepared glass knife. Sections of 300 nm thickness were placed on a 10 mm diameter As:Si-wafer and coated with a 10 nm of gold/palladium (80/20 weight ratio; Plano, Germany) conductive layer using Leica EM SCD 500 sputter-coater (Leica Microsystems, Germany) at 35 mA of Ar^+^ current against Au/Pd target kept at -0.5 kV potential.

### Maize-root ^2^H labelling and sample preparation

A long-lasting sample of resin-embedded plant root was prepared to facilitate the optimization of different ^2^H mapping approaches in this study. This was necessary because the alignment of a NanoSIMS 50L instrument for multi-isotope tracing, involving the comparison of ^2^H fraction in [^2^H/^1^H, ^12^C^2^H/^12^C^1^H, ^16^O^2^H/^16^O^1^H, ^12^C_2_^2^H/^12^C_2_^1^H] series of isotopologue ratios, takes several hours. Additionally, due to the low natural abundance of deuterium (0.0115 at%) and a relatively low yield of ^2^H containing secondary ions, the alignment and corresponding measurements of ^2^H fraction have to be performed with the current of primary Cs^+^ ions increased up to 4–6 pA implying an enhanced consumption of the sample material. Wild-type maize seeds provided by the Institute of Crop Science and Resource Conservation, University of Bonn, were first surface sterilized^34^. Plant growth involving ^2^H-labelling with 40% heavy water (^2^H_2_O, 99.8 at% ^2^H, Sigma Aldrich, Germany) was performed according to^35^. The primary 1 cm root tip was harvested after 96 h. The root fixation, LR White resin infiltration, curring and trimming have been performed in the same way as described for the OPG samples (Sect. "Mass-spectroscopy of 2H-containing ion species"). LR white block with a root crossection in its trimmed face was mounted in 10 mm diameter ring (fitting the nanoSIMS “Biology” sample holder) and coated with 10 nm of gold/palladium as OPG thin-section samples (Sect. "Mass-spectroscopy of 2H-containing ion species").

### NanoSIMS analysis and data processing

The analysis of multi-elemental isotope ratios was implemented with a serial NanoSIMS 50L #134 instrument (AMETEK, CAMECA, France) at the UFZ in Leipzig. A set of 15 secondary ion species (^1^H^−^, ^2^H^–^, ^12^C^1^H^–^, ^12^C^2^H^–^, ^16^O^1^H^–^, ^16^O^2^H^–^, ^12^C_2_^−^, ^12^C^13^C^–^, ^12^C_2_^1^H^−^, ^12^C^14^N^−^, ^12^C_2_^2^H^–^, ^12^C^15^N^−^, ^13^C^14^N^−^, ^31^P^−^, ^32^S^−^ and ^31^P^1^H^−^) were detected with seven detectors upon different mass-assignment configurations involving deflector-plate switching (see Results and Discussion for more details). Measurements were conducted with 15×120 μm (width x height) nominal size of the entrance slit, 40×1800 µm exit slits, 150×150 μm aperture and an energy slit cutting off 30% of secondary ions in their energy-distribution tail. Caesium (Cs) pre-implantation was performed with 16 keV Cs^+^ in 200 pA beam rastering within 100×100 µm^2^ area for 30 min. Within these pre-implanted areas, 15×15 µm^2^ or 25×25 µm^2^ fields of view (FoV) were scanned in a 512×512 pixel raster using a 4 pA primary Cs^+^ beam with a dwelling time of 2 ms/pixel. To ensure sufficient counting statistics, data from the same FoV were acquired over 160 scans with the deflector-plate voltages switching between two values every second scan.

Processing of the acquired data was done with a modified version of the open-source Look@NanoSIMS software^36^ (details in SI section S4). The data acquired with each scan were corrected for the lateral drift in the secondary electron intensity map (Esi) and all detected planes were accumulated for each ion species. The accumulated ^12^C^14^N^−^ map, which is a proxy of the intrinsic cellular biomarkers (Fig. S6), was then used to draw regions of interest (RoIs) corresponding to plant tissues (maize sample) or microbial cells (OPG sample). Finally, the isotope ratios in the RoI pixels were exported in a text format suitable for further statistical analysis.

## Supplementary Information

Below is the link to the electronic supplementary material.

Supplementary Material 1

## Data Availability

The datasets generated and analysed during the current study are available in the Google repository, https://drive.google.com/drive/folders/1iPocpciynqBhEu5QOU-qrUmrcPzcnpZK?usp=sharing

## References

[1] Foster, R. A., Sztejrenszus, S. & Kuypers, M. M. Measuring carbon and N_2_ fixation in field populations of colonial and free-living unicellular cyanobacteria using nanometer-scale secondary ion mass spectrometry(1). *J. Phycol.* **49**(3), 502–516 (2013). 27007039 10.1111/jpy.12057

[2] LaVan, D. A. & Cha, J. N. Approaches for biological and biomimetic energy conversion. *Proc. Natl. Acad. Sci. U S A* **103**(14), 5251–5255 (2006). 16567648 10.1073/pnas.0506694103 PMC1459341

[3] Musat, N. et al. A single-cell view on the ecophysiology of anaerobic phototrophic bacteria. *Proc. Natl. Acad. Sci. U S A* **105**(46), 17861–17866 (2008). 19004766 10.1073/pnas.0809329105 PMC2582579

[4] Lechene, C. et al. High-resolution quantitative imaging of mammalian and bacterial cells using stable isotope mass spectrometry. *J. Biol.* **5**(6), 20 (2006). 17010211 10.1186/jbiol42 PMC1781526

[5] McMahon, G., Glassner, B. J. & Lechene, C. P. Quantitative imaging of cells with multi-isotope imaging mass spectrometry (MIMS)—Nanoautography with stable isotope tracers. *Appl. Surf. Sci.* **252**(19), 6895–6906 (2006).

[6] Musat, N. et al. Detecting metabolic activities in single cells, with emphasis on nanoSIMS. *FEMS Microbiol. Rev.* **36**(2), 486–511 (2012). 22092433 10.1111/j.1574-6976.2011.00303.x

[7] Berry, D. et al. Tracking heavy water (D2O) incorporation for identifying and sorting active microbial cells. *Proc. Natl. Acad. Sci. U S A* **112**(2), E194-203 (2015). 25550518 10.1073/pnas.1420406112 PMC4299247

[8] Worrich, A. et al. Mycelium-mediated transfer of water and nutrients stimulates bacterial activity in dry and oligotrophic environments. *Nat. Commun.* **8**, 15472 (2017). 28589950 10.1038/ncomms15472 PMC5467244

[9] Mosin, O. V. & Ignatov, I. Isotope Purification of Drinking Water from Heavy Isotopes – Deuterium (2H), Tritium (3H) and Oxygen (18O). *J. Med. Physiol. Biophys.* **19**, 82–96 (2015).

[10] Zhang, X., Gillespie, A. L. & Sessions, A. L. Large D/H variations in bacterial lipids reflect central metabolic pathways. *Proc. Natl. Acad. Sci. U S A* **106**(31), 12580–12586 (2009). 19617564 10.1073/pnas.0903030106 PMC2722351

[11] Misra, P. M. The effects of deuterium on living organisms. *Curr. Sci.* **36**(17), 447–453 (1967).

[12] Tyrrell, T. Redfield Ratio. In *Encyclopedia of Ocean Sciences* (ed. Steele, J. H.) 2377–2387 (Academic Press, 2001).

[13] Kopf, S. H. et al. Heavy water and (15) N labelling with NanoSIMS analysis reveals growth rate-dependent metabolic heterogeneity in chemostats. *Environ. Microbiol.* **17**(7), 2542–2556 (2015). 25655651 10.1111/1462-2920.12752 PMC4587896

[14] Steinhauser, M. L. et al. Quantifying cell division with deuterated water and multi-isotope imaging mass spectrometry (MIMS). *Surf. Interfa. Anal.* **46**(Suppl 1), 161–164 (2014). 10.1002/sia.5581 PMC4566163 26379340

[15] Guillermier, C. et al. Imaging mass spectrometry demonstrates age-related decline in human adipose plasticity. *JCI insight* **2**(5), e90349 (2017). 28289709 10.1172/jci.insight.90349 PMC5333969

[16] Li, K. et al. NanoSIMS imaging and analysis in materials science. *Annual Rev. Anal. Chem.* **13**, 273–292 (2020). 10.1146/annurev-anchem-092019-032524 32040924

[17] Slodzian, G. et al. Simultaneous hydrogen and heavier element isotopic ratio images with a scanning submicron ion probe and mass resolved polyatomic ions. *Microsc. Microanal.* **20**(2), 577–581 (2014). 24548344 10.1017/S1431927613014074

[18] Milferstedt, K. et al. The importance of filamentous cyanobacteria in the development of oxygenic photogranules. *Sci. Rep.* **7**(1), 17944 (2017). 29263358 10.1038/s41598-017-16614-9 PMC5738420

[19] Abouhend, A. S. et al. The oxygenic photogranule process for aeration-free wastewater treatment. *Environ. Sci. Technol.* **52**(6), 3503–3511 (2018). 29505719 10.1021/acs.est.8b00403

[20] Brockmann, D. et al. Wastewater treatment using oxygenic photogranule-based process has lower environmental impact than conventional activated sludge process. *Biores. Technol.* **319**, 124204 (2021). 10.1016/j.biortech.2020.124204 33038652

[21] Guillermier, C. et al. Quantitative imaging of deuterated metabolic tracers in biological tissues with nanoscale secondary ion mass spectrometry. *Int J Mass Spectrom* **422**, 42–50 (2017). 29276427 10.1016/j.ijms.2017.08.004 PMC5739342

[22] Stryhanyuk, H. et al. Calculation of single cell assimilation rates from sip-nanosims-derived isotope ratios: A comprehensive approach. *Front Microbiol* **9**, 2342 (2018). 30337916 10.3389/fmicb.2018.02342 PMC6178922

[23] Musat, N. et al. The effect of FISH and CARD-FISH on the isotopic composition of 13C- and 15N-labeled pseudomonas putida cells measured by nanoSIMS. *Syst. Appl. Microbiol.* **37**(4), 267–276 (2014). 24702905 10.1016/j.syapm.2014.02.002

[24] Simon, R. D. Measurement of the cyanophycin granule polypeptide contained in the blue-green alga Anabaena cylindrica. *J. Bacteriol* **114**(3), 1213–1216 (1973). 4197270 10.1128/jb.114.3.1213-1216.1973 PMC285384

[25] Allen, M. M., Morris, R. & Zimmerman, W. Cyanophycin granule polypeptide protease in a unicellular cyanobacterium. *Arch. Microbiol.* **138**(2), 119–123 (1984).

[26] Cleveland, C. C. & Liptzin, D. C:N:P stoichiometry in soil: is there a “Redfield ratio” for the microbial biomass?. *Biogeochemistry* **85**(3), 235–252 (2007).

[27] Della-Negra, O. et al. History of carbon supply shapes the metabolic response of photogranules to light shifts. *Water Res.* **281**, 123557 (2025). 40156996 10.1016/j.watres.2025.123557

[28] Zhang, Z. et al. Chemical basis for deuterium labeling of fat and NADPH. *J. Am. Chem. Soc.* **139**(41), 14368–14371 (2017). 28911221 10.1021/jacs.7b08012 PMC5748894

[29] Spaans, S. K. et al. NADPH-generating systems in bacteria and archaea. *Front. Microbiol.* **6**, 742 (2015). 26284036 10.3389/fmicb.2015.00742 PMC4518329

[30] Polerecky, L. et al. Calculation and interpretation of substrate assimilation rates in microbial cells based on isotopic composition data obtained by nanoSIMS. *Front Microbiol* **12**, 621634 (2021). 34917040 10.3389/fmicb.2021.621634 PMC8670600

[31] Price, P. B. & Sowers, T. Temperature dependence of metabolic rates for microbial growth, maintenance, and survival. *Proc. Natl. Acad. Sci.* **101**(13), 4631–4636 (2004). 15070769 10.1073/pnas.0400522101 PMC384798

[32] Calabrese, F. et al. Quantitation and comparison of phenotypic heterogeneity among single cells of monoclonal microbial populations. *Front. Microbiol.* **10**, 2814 (2019). 31921014 10.3389/fmicb.2019.02814 PMC6933826

[33] Ouazaite, H. et al. Mapping the biological activities of filamentous oxygenic photogranules. *Biotechnol. Bioeng.* **118**(2), 601–611 (2021). 33006374 10.1002/bit.27585

[34] Davoudpour, Y. et al. High resolution microscopy to evaluate the efficiency of surface sterilization of Zea Mays seeds. *PLoS ONE* **15**(11), e0242247 (2020). 33253171 10.1371/journal.pone.0242247 PMC7703986

[35] Davoudpour, Y. et al. Tracking deuterium uptake in hydroponically grown maize roots using correlative helium ion microscopy and Raman micro-spectroscopy. *Plant Methods* **19**(1), 71 (2023). 37452400 10.1186/s13007-023-01040-y PMC10347822

[36] Polerecky, L. et al. Look@NanoSIMS: A tool for the analysis of nanoSIMS data in environmental microbiology. *Environ. Microbiol.* **14**(4), 1009–1023 (2012). 22221878 10.1111/j.1462-2920.2011.02681.x

