# Supplementary material for: Restoration of deuterium marker for multi-isotope mapping of cellular metabolic activity
Source: Sci Rep. 2026 Jan 7;16:883. doi: 10.1038/s41598-025-33762-5 (PMC12783644; doi:10.1038/s41598-025-33762-5)
Supplement: Supplementary file 1 — Supplementary Material 1 [file 41598_2025_33762_MOESM1_ESM.pdf]

Supplementary information

Restoration of deuterium marker for multi-isotope mapping of cellular metabolic activity

Nadiia Yamborko[1,2,3], Laura Schwab[1,a], Lubos Polerecky[4], Yalda Davoudpour[1,b], Hugo Berthelot[1,c], Niculina Musat[1,d], Kim Milferstedt[5], Jérôme Hamelin[5], Hans-Hermann Richnow[1,e], Carsten Vogt[1], Hryhoriy Stryhanyuk[1,*]

[1] *Department of Technical Biogeochemistry, Helmholtz Centre for Environmental Research – UFZ, Permoserstraße 15, 04318 Leipzig, Germany*

[2] *Department of Soil Ecology, Helmholtz Centre for Environmental Research – UFZ, Theodor-Lieser-Straße 4, 06120 Halle, Germany*

[3] *Department of General and Soil Microbiology, Zabolotny Institute of Microbiology and Virology, NASU, Akademik Zabolotniy's Str. 154, 03680 Kyiv, Ukraine*

[4] *Department of Earth Sciences, Faculty of Geosciences, Utrecht University, Utrecht, The Netherlands*

[5] *INRAE, University Montpellier, LBE, 102 Avenue des étangs, F-11000 Narbonne, France*

***Corresponding author:** gregory.stryhanyuk@ufz.de*

Current Affiliation:

[a]L.S.: Institute of Biodiversity, Aquatic Geomicrobiology, Friedrich Schiller University Jena, Dornburgerstraße 159, 07743 Jena, Germany

[b]Y.D.: Institute of Inorganic and Materials Chemistry, University of Cologne, Greinstraße 6, 50939 Cologne, Germany

[c]H.B.: IFREMER, DYNECO, Pelagos Laboratory, 29280 Plouzané, France

[d]N.M.: Department of Biology, Section for Microbiology, Aarhus University, Ny Munkegade 114, 8000 Aarhus C, Denmark

[e]H.-H.R.: ISODETECT Leipzig, Deutscher Platz 5b, 04103 Leipzig, Germany

S1. Multicollector settings

High-resolution nanoSIMS analysis of cellular deuterium fractions with a single-atomic $^{2}H/^{1}H$ isotope ratio may involve parallel acquisition of carbon $^{13}C/^{12}C$ isotope ratio, but does not allow for simultaneous tracing of nitrogen assimilation. The reason for this limitation is a construction feature of the NanoSIMS 50L instrument restricting the ratio of maximal to minimal mass measured simultaneously as M_{max}/M_{min}=22. A shift of the factory-set end-position for $^{1}H^{-}$ counting detector (detector#1) in low-mass direction may help to detect $^{1}H^{-}$ with an increased magnetic field-strength and therefore higher M_{max}, bringing the mass-range ratio up to M_{max}/M_{min}=28 [Kopf 2015]. Another strategy of multi-isotope tracing implies the deriving of $^{2}H/^{1}H$ isotope ratio from polyatomic ions, i.e., with $^{12}C^{2}H/^{12}C^{1}H$, $^{16}O^{2}H/^{16}O^{1}H$ or $^{12}C_2{}^{2}H/^{12}C_2{}^{1}H$ isotopologue ratios. In this way, the constraints imposed by the M_{max}/M_{min} ratio are released and the measuring at 27 amu becomes accessible for tracing of nitrogen ($^{12}C^{15}N/^{12}C^{14}N$) and carbon ($^{13}C^{14}N/^{12}C^{14}N$) metabolization into the biomass. Extended in this way, the mass range allows for multi-isotope tracing of H, C and N assimilation as well as for the mapping of important macro-, meso- (e.g., ^{31}P, ^{32}S) and microelements (e.g., ^{63}Cu, ^{80}Se, ^{127}I). Nevertheless, deuterium detection in polyatomic $^{12}C^{2}H^{-}$, $^{16}O^{2}H^{-}$ and $^{12}C_2{}^{2}H^{-}$ ion species requires high mass-resolving power (MRP=M/dM) of about 9.000-17.000 [Slodzian 2014] to separate them from interfering isobars that are $^{13}C^{1}H^{-}$ & $^{12}C^{1}H_2{}^{-}$, $^{17}O^{1}H^{-}$ & $^{16}O^{1}H_2{}^{-}$ and $^{12}C^{13}C^{1}H^{-}$ & $^{12}C_2{}^{1}H_2$, respectively. An upgrade of NanoSIMS 50L involving the reduction of the entrance-slit width to 5 µm and the exit-slit width down to 15-20 µm enables clear separation of $^{12}C^{2}H$, $^{16}O^{2}H$ and $^{12}C_2{}^{2}H$ mass-peaks with MRP=25.000 [Slodzian 2014]. Otherwise, employing a serial NanoSIMS 50L instrument with the smallest exit slit of 40 µm width, it is challenging up to impossible to resolve the interferences with ^{2}H containing polyatomic ions mentioned above. When the instrument upgrade is not possible, a numerical restoration may also solve the unresolved interference issue. For the restoration of $^{16}O^{2}H^{-}$ ion counts, it was suggested to resolve the $^{17}O^{1}H$ & $^{16}O^{2}H$ interference via subtracting $^{16}O^{1}H$ counts multiplied by $^{17}O/^{16}O$ isotope ratio [Berry 2015].

Seven detectors available with the employed NanoSIMS 50L instrument were set in several configurations to detect four pairs of hydrogen-containing secondary ion species (Table S1, Settings I-III) and infer the feasibility of simultaneous multi-element tracing of H, C and N isotope ratios (Setting IV). Analyses involving the detection of different ion species with the same detector, such as $^{12}C^{14}N^{-}$ & $^{12}C_2{}^{2}H^{-}$ and $^{12}C^{15}N^{-}$ & $^{13}C^{14}N^{-}$ in Setting IV, were implemented via switching the deflection

voltage (setting deflection voltage for neighbour mass-peaks with e.g., HMR-Scan routine) while keeping the magnetic field strength fixed in the „Magnetic Peak-switching“ mode.

In general, H^- detection requires check and readjustment of correction magnetic coils to adjust the Cy and P2/P3 scan profiles of H^- ions with those for heavier ions. This is especially crucial when analysing slanted sample area, i.e., samples with “non-ideal” flatness or conductivity and areas close to the sample edge.

Table S1: Settings of the NanoSIMS 50L multi-collector unit for ^{2}H and multi-isotope tracing.

**Detector #**	**Secondary ion species**				
	**Setting I**	**Setting II**	**Setting III**	**Setting IV**	**Setting V**
**1**	$^{2}H^{-}$	$^{1}H^{-}$	$^{1}H^{-}$	$^{1}H^{-}$	$^{16}O^{1}H^{-}$
**2**	$^{12}C^{1}H^{-}$	$^{2}H^{-}$	$^{2}H^{-}$	$^{2}H^{-}$	$^{16}O^{2}H^{-}$
**3**	$^{12}C^{2}H^{-}$	$^{12}C^{1}H^{-}$	$^{12}C^{1}H^{-}$	$^{12}C^{1}H^{-}$	$^{12}C_2^{-}$
**4**	$^{16}O^{1}H^{-}$	$^{12}C^{2}H^{-}$	$^{12}C^{2}H^{-}$	$^{12}C^{2}H^{-}$	$^{12}C^{13}C^{-}$ & $^{12}C_2{}^{1}H^{-}$
**5**	$^{16}O^{2}H^{-}$	$^{16}O^{1}H^{-}$	$^{16}O^{1}H^{-}$	$^{12}C_2{}^{1}H^{-}$	$^{12}C^{14}N^{-}$ & $^{12}C_2{}^{2}H^{-}$
**6**	$^{12}C_2{}^{1}H^{-}$	$^{16}O^{2}H^{-}$	$^{12}C_2{}^{1}H^{-}$	$^{12}C^{14}N^{-}$ & $^{12}C_2{}^{2}H^{-}$	$^{12}C^{15}N^{-}$ & $^{13}C^{14}N^{-}$
**7**	$^{12}C_2{}^{2}H^{-}$	$^{12}C^{14}N^{-}$	$^{12}C_2{}^{2}H^{-}$	$^{12}C^{15}N^{-}$ & $^{13}C^{14}N^{-}$	$^{32}S^{-}$ & $^{31}P^{1}H^{-}$
**Comments**	factory settings	det#1 end-switch shifted towards low-mass			factory settings
	M_{min}=2 amu	M_{min}=1 amu			M_{min}=17 amu
	M_{max}=26 amu	M_{max}=26 amu		M_{max}=27 amu	M_{max}=32 amu

The shift of the end-switch position for trolley #1 towards low-mass [Kopf 2015] allowed us to catch ^{1}H in detector #1 simultaneously with mass 27 amu ($^{12}C^{15}N^{-}$ or $^{13}C^{14}N^{-}$) in detector #7 (Settings IV, Table S1). However, the count rate of $^{1}H^{-}$ ions was found to be reduced in this configuration of multicollection. As a possible reason for the observed reduction in the $^{1}H^{-}$ count rate, one may consider the increase in the magnetic field strength upon change from 26 amu to 27 amu in detector #7. The increased strength of magnetic stray field may cause a deflection of $^{1}H^{-}$ ions as they approach the magnet after leaving the SS100 electrostatic analyser. This deflection of $^{1}H^{-}$ ions may be revealed by observing considerably different trends in the count rates of H^{-} and heavier ions upon changing the energy-slit position (EnS.X). Thus, the extension of the mass-range by shifting the end-switch of detector #1 may not be favourable for all NanoSIMS 50L instruments. For multi-isotope tracing involving $M_{max} > 26$ amu, one may consider avoiding $^{1}H^{-}$ detection if CH^{-} or C_2H^{-} are available

for the quantitation of ^{2}H fraction. Molecular-specific (e.g., sugar) ^{2}H enrichment may be derived from the $^{16}O^2H^-/^{16}O^1H^-$ ratio. However, one needs to consider and check for the spontaneous (abiotic) ^{1}H-^{2}H exchange in OH groups.

S2. Restoration of ^2H fraction from the unresolved $^{12}C_2{}^2H^-$ & $^{12}C_2{}^1H_2^-$ mass-peak

In a biological system, the assimilation of ^{2}H-enriched growth substrate leads to an increase in the cellular $^2\mathrm{H}$ fraction, $F(^2\mathrm{H}) = {}^2\mathrm{H}/(^2\mathrm{H} + {}^1\mathrm{H})$. This change in the isotopic composition is reflected statistically well (i.e., with good counting statistics) by the secondary ions of molecular fragments $\mathrm{C_2H}$ and $\mathrm{C_2H_2}$. The 16 keV primary Cs$^+$ ion beam in a nanoSIMS experiment causes $^{12}\mathrm{C_2}{}^1\mathrm{H_2}^-$ to be probably the largest, but still well abundant, secondary ion representing fragmented biomolecules. Although the high counting rate of $^{12}\mathrm{C_2}{}^2\mathrm{H}^-$ ion species offers an enhanced precision in the quantitation of $^2\mathrm{H}$ fraction, its accuracy is strongly corrupted by the contribution of $^{12}\mathrm{C_2}{}^1\mathrm{H_2}^-$ ion counts into the unresolved $^{12}\mathrm{C_2}{}^2\mathrm{H}$ & $^{12}\mathrm{C_2}{}^1\mathrm{H_2}$ mass-peak (Fig. S1).

The ion species contributing to the unresolved $^{12}\mathrm{C_2}{}^2\mathrm{H}$ & $^{12}\mathrm{C_2}{}^1\mathrm{H_2}$ peak contain either light ($^1\mathrm{H}$) or heavy ($^2\mathrm{H}$) hydrogen isotope. We denote the fraction of $^{12}\mathrm{C_2}{}^1\mathrm{H_2}^-$ additives by

$$F^{Add} = {}^{12}\mathrm{C_2}{}^1\mathrm{H_2}/({}^{12}\mathrm{C_2}{}^2\mathrm{H} + {}^{12}\mathrm{C_2}{}^1\mathrm{H_2}) \quad \text{(Eq. S1)}$$

and the corresponding ratio by

$$R^{Add} = {}^{12}\mathrm{C_2}{}^1\mathrm{H_2}/{}^{12}\mathrm{C_2}{}^2\mathrm{H}. \quad \text{(Eq. S2)}$$

Additionally, we denote the true ^{2}H fraction in the sample by

$$F(^2\mathrm{H}) \equiv F = {}^{12}\mathrm{C_2}{}^2\mathrm{H}\,/({}^{12}\mathrm{C_2}{}^1\mathrm{H} + {}^{12}\mathrm{C_2}{}^2\mathrm{H}), \quad \text{(Eq. S3)}$$

and the corresponding true ^{2}H/^{1}H ratio by

$$R(^2\mathrm{H}) \equiv R = {}^{12}\mathrm{C_2}{}^2\mathrm{H}\,/{}^{12}\mathrm{C_2}{}^1\mathrm{H} \; = F/(1-F). \quad \text{(Eq. S4)}$$

Equation S1 reveals that the contribution of $^{12}\mathrm{C_2}{}^1\mathrm{H_2}^-$ is expected to decrease upon increasing $^2\mathrm{H}$ fraction, as previously noted by [Guillermier 2017] and illustrated in Fig. S1. Quantitative validation of this quite justified expectation is demonstrated by the following series of analytical transformations.

Because of the unresolved interference of $^{12}\mathrm{C_2}{}^2\mathrm{H}^-$ with $^{12}\mathrm{C_2}{}^1\mathrm{H_2}^-$ additives, the measured ion counts result in the overestimated deuterium isotope ratio:

$$R' = (^{12}\mathrm{C}_2{}^2\mathrm{H} + {}^{12}\mathrm{C}_2{}^1\mathrm{H}_2) / {}^{12}\mathrm{C}_2{}^1\mathrm{H} . \qquad \text{(Eq. S5)}$$

For the restoration of $^2\mathrm{H}$ fraction with the ion counts in the unresolved $^{12}\mathrm{C}_2{}^2\mathrm{H}$ & $^{12}\mathrm{C}_2{}^1\mathrm{H}_2$ mass-peak, we will now derive a relationship between R' and R. The key assumptions in our derivation are that (i) the ion yield for a specific polyatomic ion only depends on the atomic composition of the ion and not on its isotopic composition, and (ii) the distribution of isotopes within the possible isotopologues of the polyatomic fragment is random. The second assumption means that the probability of selecting an isotope ^mX from the pool of X is equal to its fraction within the pool, $F(^mX)$. Thus, for a specific polyatomic ion $X_aY_b{}^-$, we assume that the number of detected isotopologue ions $^mX_a{}^nY_b{}^-$ is given by the product of the overall polyatomic ion yield (one value for the entire isotopologue series), denoted as X_aY_b, and the probability of the specific isotope combination, which is equal to the product $[F(^mX)]^a \times [F(^nY)]^b$. Overall, the key assumption in our derivation is formalized by the following expression for the number of detected isotopologue ions:

$$^mX_a{}^nY_b = X_aY_b \times [F(^mX)]^a \times [F(^nY)]^b \qquad \text{(Eq. S6)}$$

Note that the molecular ion yield X_aY_b is independent of the isotope mass (*m* and *n*) as emphasized above.

Next, we apply Eq. S6 to the target isotopologue ions $^{12}\mathrm{C}_2{}^1\mathrm{H}^-$ and $^{12}\mathrm{C}_2{}^2\mathrm{H}^-$ and the interfering ion $^{12}\mathrm{C}_2{}^1\mathrm{H}_2{}^-$ involved in the calculation of R (Eq. S4) and R' (Eq. S5). According to Eq. S6, the number of detected ions is expressed as follows:

$$\begin{aligned} {}^{12}\mathrm{C}_2{}^1\mathrm{H} &= C_2H \times [1 - F(^{13}\mathrm{C})]^2 \times [1 - F(^2\mathrm{H})] \\ {}^{12}\mathrm{C}_2{}^2\mathrm{H} &= C_2H \times [1 - F(^{13}\mathrm{C})]^2 \times F(^2\mathrm{H}) \qquad , \\ {}^{12}\mathrm{C}_2{}^1\mathrm{H}_2 &= C_2H_2 \times [1 - F(^{13}\mathrm{C})]^2 \times [1 - F(^2\mathrm{H})]^2 \end{aligned} \qquad \text{(Eqs. S7)}$$

where C_2H and C_2H_2 denote the overall yield of the corresponding $\mathrm{C_2H^-}$ and $\mathrm{C_2H_2{}^-}$ polyatomic ions, $F(^{13}\mathrm{C})$ and $[1 - F(^{13}\mathrm{C})]$ denote the fraction of the ^{13}C and ^{12}C isotopes, and $F(^2\mathrm{H})$ and $[1 - F(^2\mathrm{H})]$ denote the fraction of the ^{2}H and ^{1}H isotopes, respectively.

With expressions Eqs. S7, we combine Eqs. S4 and S5 to obtain the following relationship between R' and R:

$$R' = \frac{^{12}\mathrm{C}_2{}^2\mathrm{H}}{^{12}\mathrm{C}_2{}^1\mathrm{H}} + \frac{^{12}\mathrm{C}_2{}^1\mathrm{H}_2}{^{12}\mathrm{C}_2{}^1\mathrm{H}} = R + \frac{^{12}\mathrm{C}_2{}^1\mathrm{H}_2}{^{12}\mathrm{C}_2{}^1\mathrm{H}} \times \underbrace{\frac{^{12}\mathrm{C}_2{}^2\mathrm{H}}{^{12}\mathrm{C}_2{}^2\mathrm{H}}}_{=1} = R + R \times \frac{^{12}\mathrm{C}_2{}^1\mathrm{H}_2}{^{12}\mathrm{C}_2{}^2\mathrm{H}}.$$

Taking into account Eq. S2, this relationship between R' and R can be expressed as:

$$R' = R \times (1 + R^{Add}). \qquad \text{(Eq. S8)}$$

As expected, the apparent value of the deuterium-to-hydrogen ratio R' increases relative to the true ratio R as a consequence of the unresolved interference with the $^{12}C_2{}^1H_2{}^-$ ion species. Note that the value of R^{Add} corresponds directly to the relative difference between R' and R, when expressed in the commonly used δ-notation, i.e., $R^{Add} = R'/R - 1 \equiv \delta^{Add}$.

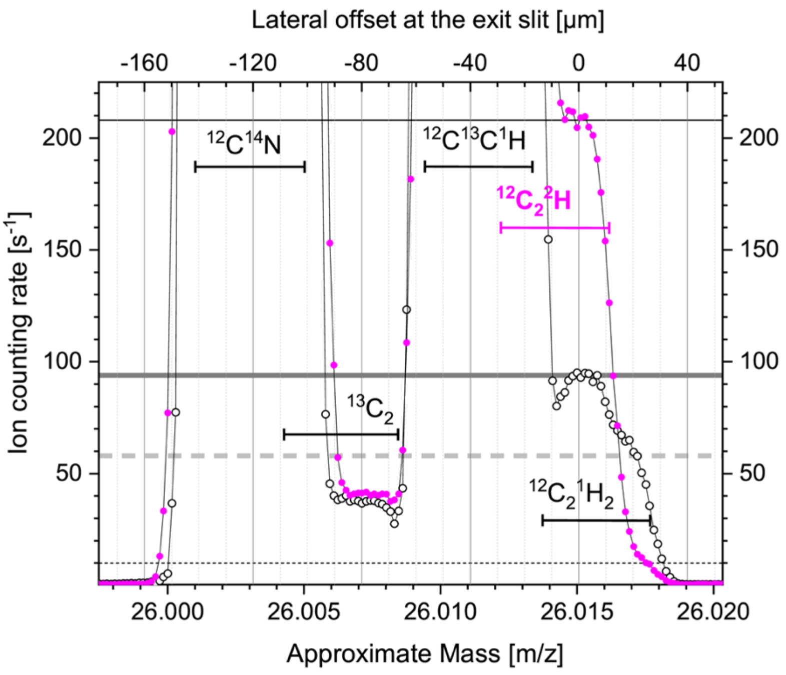

Fig. S1. High-resolution scan of the 26 amu mass peak showing the overlap and contribution of unresolved $^{12}C_2{}^2H^-$ and $^{12}C_2{}^1H_2{}^-$ ion species at natural ^2H abundance (hollow-circled) and in the sample enriched in ^2H (solid-circled). The $^{12}C_2{}^1H_2{}^-$ count rate decreases from ≈60 cps (dashed horizontal line) down to ≈10 cps (dotted horizontal line) when changing from the natural abundance to about 0.8 at% of ^2H .

The expression of R^{Add} (Eq. S2) can be rewritten by introducing the expressions for the corresponding ion counts from Eqs. S7:

$$R^{Add} = \frac{C_2H_2}{C_2H} \times \frac{[1-F(^2\mathrm{H})]^2}{F(^2\mathrm{H})}\,. \qquad \text{(Eq. S9)}$$

As we can see, R^{Add} depends only on the C_2H_2/C_2H ratio, which we hereafter denote by $\epsilon_{H_2/H}$, and the true deuterium fraction $F(^2\mathrm{H})$. With the $F(^2\mathrm{H})$ fraction expressed in terms of the hydrogen isotope ratio R (Eq. S3), R^{Add} expression (Eq. S9) is rewritten as

$$R^{Add} = \epsilon_{H_2/H} \times \frac{1}{R\cdot(R+1)}. \qquad \text{(Eq. S10)}$$

Finally, we introduce the obtained R^{Add} expression (Eq. S10) into Eq. S8, which yields the following compact expression for the relationship between R' and R:

$$R' = R + \frac{\epsilon_{H_2/H}}{1+R}. \qquad \text{(Eq. S11)}$$

To recover R from R', one needs to know the value of $\epsilon_{H_2/H}$. This value can be obtained by determining R' in a sample with a known isotopic composition, i.e., a known R value. The best approach to do this is to use a sample with a natural deuterium abundance. For the non-labelled maize-root sample, the natural hydrogen isotope ratio R_0=(1.3202±0.4500) × 10^{-4} and the apparent $R_0{}'$=(2.9609±0.3400) × 10^{-4} ratio were determined with the measured ion-counts as $^{12}\mathrm{C}^2\mathrm{H}/^{12}\mathrm{C}^1\mathrm{H}$ and $(^{12}\mathrm{C}_2{}^1\mathrm{H}_2 + {}^{12}\mathrm{C}_2{}^2\mathrm{H})/^{12}\mathrm{C}_2{}^1\mathrm{H}$ accordingly (see section 2.2, Fig. 2, in the main text). With these R_0 and $R_0{}'$ values and 2.3 × 10^5 as a number of analysed pixels, the $\epsilon_{H_2/H}$ has been derived by rearranging Eq. S11:

$$\epsilon_{H_2/H} = (R_0' - R_0) \times (1 + R_0) = (1.6411 \pm 0.0012) \times 10^{-4}. \qquad \text{(Eq. S12)}$$

With this value of $\epsilon_{H_2/H}$ we can predict the effect of the interfering signal from the $^{12}\mathrm{C}_2{}^1\mathrm{H}_2{}^-$ ion species as follows. First, for the deuterium fraction F in the range between 0.00001 and 1 (i.e., between 0.001 and 100 at%), we calculate the corresponding deuterium-to-hydrogen ratio R according to Eq. S4. With these R values, R^{Add} and R' are calculated using Eqs. S10 and S5, respectively. Finally, according to Eq. S3, we calculate the fraction of the $^{12}\mathrm{C}_2{}^1\mathrm{H}_2{}^-$ additives F^{Add} and the apparent deuterium fraction F'. In this way, we elucidate the dependence of the interfering $^{12}\mathrm{C}_2{}^1\mathrm{H}_2$ additive fraction F^{Add} and the overestimated F' on the deuterium fraction F (Fig. S2).

The results show that the overestimation of the $^2\mathrm{H}$ fraction is quite substantial at deuterium abundances close to the natural abundance and becomes negligible at abundances above 1 at% (compare thick solid blue and dashed black lines in Fig. S2). For example, at the natural deuterium abundance level of F_0=0.0132 at%, the apparent value is F_0'=0.0296 at%, i.e., a relative difference of more than 120%. This corresponds to an apparent $\delta'(^2\mathrm{H})$ value of about 1240‰ for a sample with

the true $\delta'(^{2}\mathrm{H})$ value of 0‰. The relative difference decreases below 5% when the deuterium enrichment exceeds 0.32 at%.

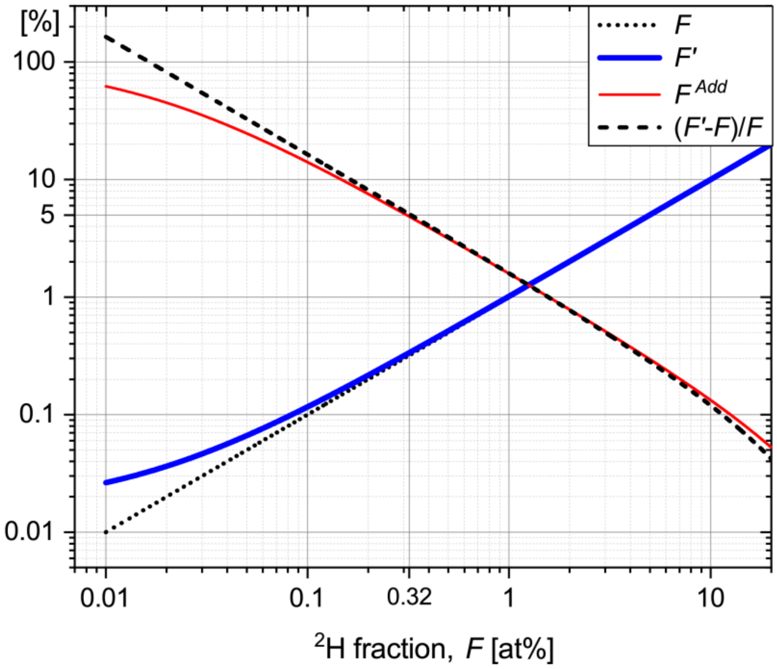

Fig. S2. Development of the additive fraction F^{Add} (Eq. 1, thin solid red line) and the apparent ^{2}H abundance F' (thick solid blue line) on the true ^{2}H abundance F. The deviation of F' from F is a consequence of the unresolved $^{12}\mathrm{C}_2{}^{2}\mathrm{H}$ & $^{12}\mathrm{C}_2{}^{1}\mathrm{H}_2$ mass-peak.

After this analysis, we can now turn to the restoration of the true hydrogen isotope ratio R from the apparent value R'. This is achieved by solving Eq. S11 for R, which can be done at various levels of accuracy, as explained below. To obtain the exact solution, we multiply Eq. S11 by $(1+R)$, rearrange the terms and obtain the following quadratic equation for R:

$$R^2 + (1 - R') \times R + \left(\epsilon_{H_2/H} - R'\right) = 0. \qquad \text{(Eq. S13)}$$

The solutions of Eq. S13 are

$$R = \frac{1-R'}{2} \times \left(-1 \pm \sqrt{1+x}\right), \qquad x = 4 \times \frac{R' - \epsilon_{H_2/H}}{(1-R')^2}. \qquad \text{(Eq. S14)}$$

Since the restored R_r can only be a positive number, the sign + or − is chosen such that the solution obtained with Eq. S14 is positive. This change of signs occurs when the apparent hydrogen isotope ratio R' changes from R'< 1 to R'> 1, i.e., when the corresponding fraction changes from F'< 0.5 to F'> 0.5.

In practical cases involving deuterium tracing, values of the hydrogen isotope ratio are very much lower than 1, i.e., $R \lll 1$. Thus, we can approximate $1/(1+R)$ in Eq. S11 by 1, which yields

$$R_r \approx R' - \epsilon_{H_2/H}\ , \qquad R \lll 1\ . \qquad \text{(Eq. S15)}$$

In situations when the deuterium enrichment is somewhat larger but still small, i.e., when $R \ll 1$, it is more appropriate to apply the approximation with a first-order term of the Taylor-series, i.e., $1/(1+R) \approx 1 - R$, which yields

$$R_r \approx \frac{R' - \epsilon_{H_2/H}}{1 - \epsilon_{H_2/H}}\ , \qquad R \ll 1\ . \qquad \text{(Eq. S16)}$$

Note that R_r approximations in Eqs. S15 and S16 are sufficiently accurate if the value of $\epsilon_{H_2/H}$ itself is very small, as in the present study. In case none of the above approximations is acceptable, the exact solution in Eq. S14 should be used to restore R from R'.

Finally, we show that the R recovered from R' with the exact solution of Eq. S14 and R_r values derived with approximations according to Eq. S15 and Eq. S16 yield almost indistinguishable values of the recovered deuterium fraction F and F_r. As shown in Fig. S3, when Eq. S15 is used ($R \lll 1$, thick lines), the absolute difference $|F_r - F|$ between the recovered and true deuterium abundance (thick dashed blue line) remains below 0.0025 at% over the entire range of F and decreases below 1.6×10^{-4} at% when $F < 1$ at%. Reconstruction according to Eq. S15 yields the relative difference $|F_r - F|/F$ remaining at around 1.6×10^{-2} % for F up to 10 at% and decreasing rapidly thereafter (thick solid black line in Fig. S3).

Application of Eq. S16 yields more accurate restoration at deuterium fraction below 50 at% ($R \ll 1$, thin lines in Fig. S3). For example, the absolute difference $|F_r - F|$ decreases below 1.6×10^{-6} at% for $F < 1$ at% when Eq. S16 is employed (thin dashed red line in Fig. S3), while the relative difference decreases progressively below 1.5×10^{-3} % as F decreases below 10 at% (thin solid black line in Fig. S3). In both cases, these numerical simulations show that the deviations of the restored (F_r) from the real $^{2}\mathrm{H}$ fraction (F) are negligible. Thus, we conclude that both suggested approximations (Eqs. S15 and S16) are sufficiently accurate in most practical applications, especially when the true $^{2}\mathrm{H}$ fractions are relatively low (<1 at%).

We emphasize that the contribution of the $^{12}\mathrm{C}_2{}^{1}\mathrm{H}_2$ additive into the unresolved $^{12}\mathrm{C}_2{}^{2}\mathrm{H}$ & $^{12}\mathrm{C}_2{}^{1}\mathrm{H}_2$ peak (R^{Add}), which is quantified by the parameter $\epsilon_{H_2/H}$, may differ for different types of organic

(biological) samples and even depending on the sample preparation method. Thus, in practical application of the restoration method above, the value of this parameter needs to be obtained via independent measurements. As done in this study, the most convenient approach to do this is by measuring $R_0' = [^{12}\mathrm{C}_2{}^2\mathrm{H} + {}^{12}\mathrm{C}_2{}^1\mathrm{H}_2]/{}^{12}\mathrm{C}_2{}^1\mathrm{H}$ and $R_0 = {}^{12}\mathrm{C}^2\mathrm{H}/{}^{12}\mathrm{C}^1\mathrm{H}$ ratios in a sample with the natural $^2\mathrm{H}$ abundance and then calculating $\epsilon_{H_2/H}$ according to Eq. S12.

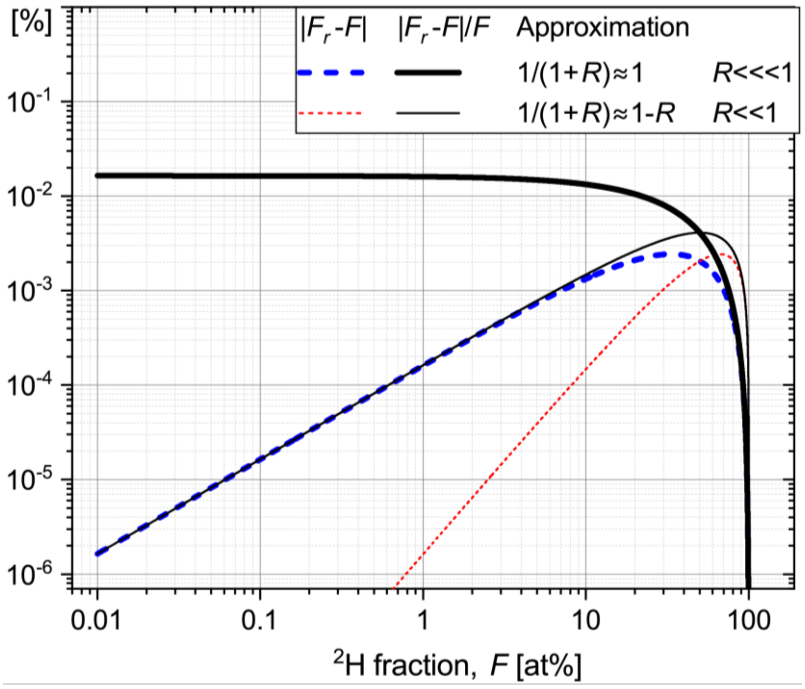

Fig. S3. Dependence of the absolute $|F_r - F|$ [at%] and relative $|F_r - F|/F$ [%] restoration error on the true $^2\mathrm{H}$ fraction, F. Results are shown for two cases when Eq. S15 ($R \lll 1$) or Eq. 16 ($R \ll 1$) is employed for the restoration.

Molecular site-specificity of $^2\mathrm{H}$-labelling is not considered in the suggested restoration of $^2\mathrm{H}$ isotope fraction (Eqs. S14-S16), but those molecular-site effects are expected to be much suppressed due to strong molecule fragmentation upon 16 keV Cs^+ ion probing in nanoSIMS experiment.

S3. Smearing effect in ^{2}H maps derived from single- and polyatomic isotopologues

Deuterium fraction mapping on a ^{2}H labelled sample of an embedded maize root shows a kind of “noisy background”. Initially, we ascribed this observation to poor counting statistics. However,

image-data acquisition from the same Field of View (FoV) in 3 subsequent series (with 160 scans each) facilitates the understanding of another possible reason for this background noise. Fig. S4 shows maps of log-scaled ^{2}H fraction derived from hydrogen isotope ratio in H, CH, OH and C_2H isotopologue pairs. The noise does not appear over the entire analysis area, but is pronounced mostly in the vicinity of ^{2}H enriched root compartments/fragments. Additionally, the noise level decreases as the mass in the [H, CH, OH, C_2H] isotopologue series increases.

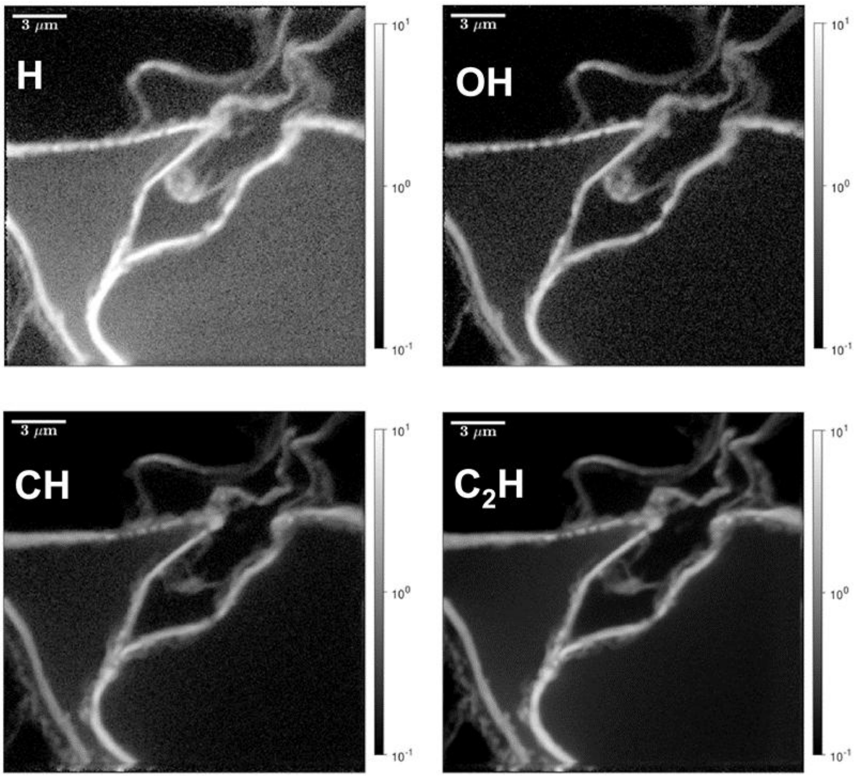

Fig. S4. Maps in the cortex zone of a maize root showing the log-scaled ^{2}H fraction derived from the hydrogen isotope ratio in H, CH, OH and C_2H isotopologue pairs.

This mass dependency is further revealed in the histograms of pixel distribution in ^{2}H fraction appeared to be quite different when derived from the different isotopologue pairs (Fig. S5 a). Dose-trends in pixel ^{2}H histograms for H and CH (Fig. S5 b,c) elucidate the effect of value averaging which is possible in case of material relocation and mixing. This relocated sample-material undergoes repetitive milling with the primary ion beam and its ionisation more probably yields small-size H-containing ion species, i.e., H^{-}, CH^{-}, OH^{-}. The relocated material might include the hydroxyl -OH groups, which are easily detached from all kinds of mono- & poly-saccharides, poly-phenols,

carboxylic acids and their derivatives upon the primary ion-beam impact. Consequently, this material-smearing effect is strongly revealed in ^{2}H maps derived from H and OH isotopic ratios (Fig. S4), it is much weaker for CH case and almost not detectable when C_2H isotopologues are considered for the restoration of the ^{2}H fraction (Fig. S5 d).

The effect of ion-beam induced material relocation may also be observed in ^{15}N-distribution maps on e.g., microbial cells; however, it is not easy to recognize any relocated material when a homogeneous sample is analysed [Bardin 2015]. The extent of sample-material relocation upon ion-probe impact may depend on ionization threshold of the detected ion species, partitioning of primary-ion energy over kinetic energy of ablated particle and ionization energy, analysed material composition (e.g., on the type of embedding material) and on analysis conditions like the current of primary ion beam, dwelling time, raster density, analysed area size. All of these factors are worth of further investigation since the revealed material-transfer effects may be reduced via, e.g., optimization of the embedding material, adjustment to the probe current and dwelling time.

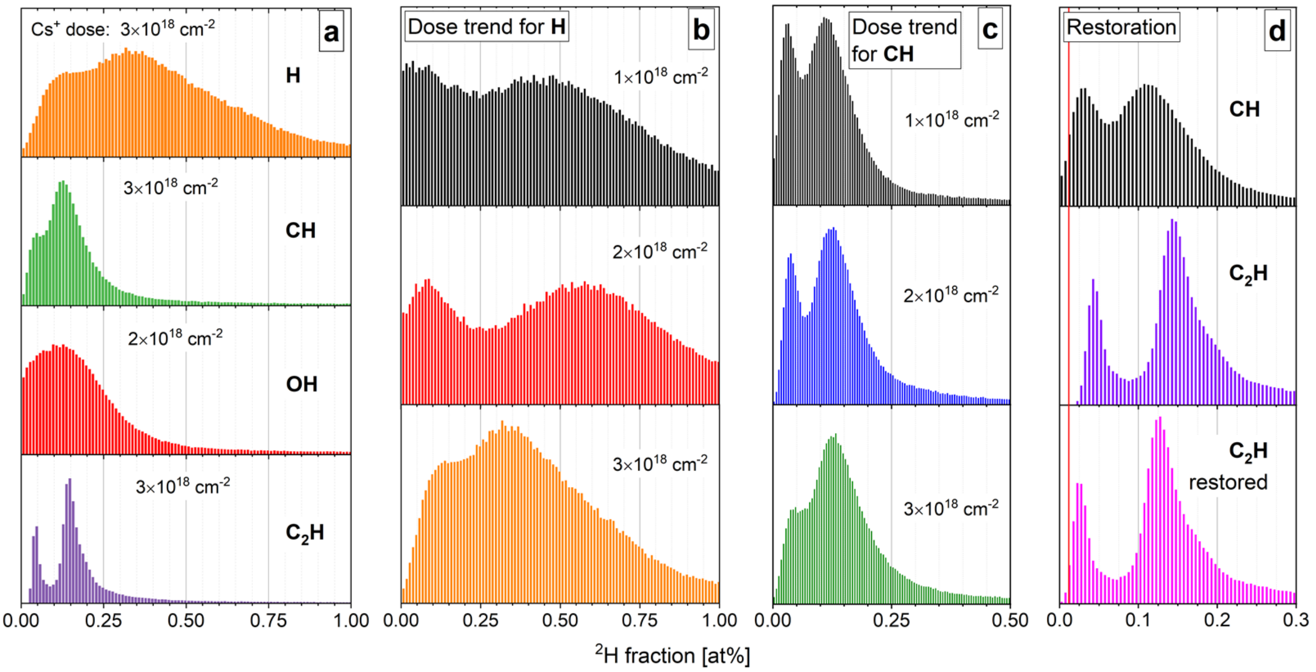

Fig. S5. Primary-ion dose dependence of pixel distribution histograms in ^{2}H fraction derived from the hydrogen isotopic ratio in H, CH, OH and C_2H isotopoloque pairs. (a) Dependence of ^{2}H fraction distribution on isotopologue pairs with the maximal dose of primary Cs^+ ions achieved. Dose trends for H isotopic ratio (b) and for CH isotopoloques (c). (d) Restoration and comparison of ^{2}H fraction from the unresolved $^{12}C_2{}^2H$ & $^{12}C_2{}^1H_2$ peak. The natural ^{2}H abundance (0.0115 at%) is shown with the vertical red line in frame (d).

S4. Enhancement in the Look@NanoSIMS software

The Look@NanoSIMS software [Polerecky 2012] was enhanced to allow the loading and processing of ion counts from up to 16 ion species at once, as opposed to a maximum of 8 ion species in the original version. Because the measurements involved the so-called peak switching, isobaric ion pairs with the same nominal mass but different, though very close, exact mass (e.g., $^{12}C^{13}C^-$ and $^{12}C_2{}^1H^-$; $^{12}C^{14}N^-$ and $^{12}C_2{}^2H^-$; $^{12}C^{15}N^-$ and $^{13}C^{14}N^-$) were detected in consecutive scans, whereas ions detected without the peak switching (e.g., $^{12}C_2{}^-$) were detected in every scan, i.e., twice a plane. Although this switching between masses is accounted for automatically by the software, special care must be taken when entering expressions for calculating isotope ratios. For example, without peak switching, the correct way to calculate the $^{13}C/^{12}C$ isotope ratio from the measured counts of $^{12}C_2{}^-$ and $^{12}C^{13}C^-$ ions is $0.5\times{}^{12}C^{13}C/^{12}C_2$. However, if peak switching is used to detect $^{12}C^{13}C^-$ and $^{12}C_2{}^1H^-$ in alternating planes, the correct way to calculate the $^{13}C/^{12}C$ isotope ratio is $^{12}C^{13}C/^{12}C_2$. Another issue arising from the peak-switching detection is that although all detected masses are automatically loaded by the software, only the first eight masses are displayed in the dedicated text fields (within the panel called “Masses”). To display these “hidden” masses, one needs to enter their name in the text field dedicated to isotope ratios (within the panel called “Ratios”), type the scale in the corresponding text field, and press “Enter”. After accounting for these peculiarities, the rest of the data processing was the same as if the loaded dataset contained the usual number of masses (eight or less).

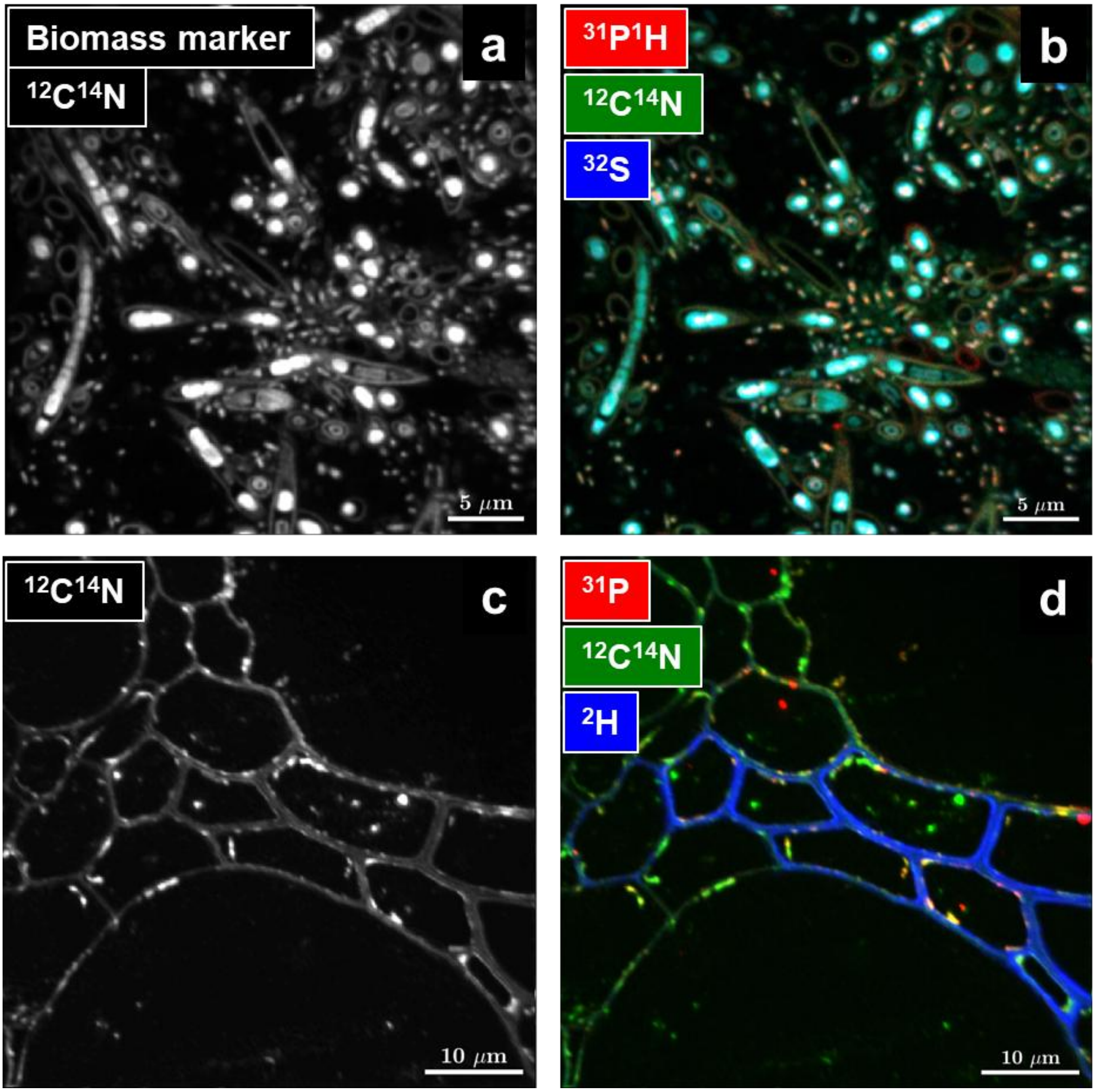

Fig. S6. $^{12}C^{14}N^{-}$ maps as a proxy of the intrinsic biomass marker acquired on microbial cells (oxygenic photogranule, frame a) and plant tissues (maize-root sample, frame c) shown in grey scale according to the ion counts acquired per pixel. Examples of possible RGB-overlays (Red, Green, Blue) in frames b and d show the relative location of phosphorus- and sulfur- reach biomass compartments in the OPG-sample (b) and the distribution of freshly-synthesized ^{2}H labellen compounds in the maize-root section (d).

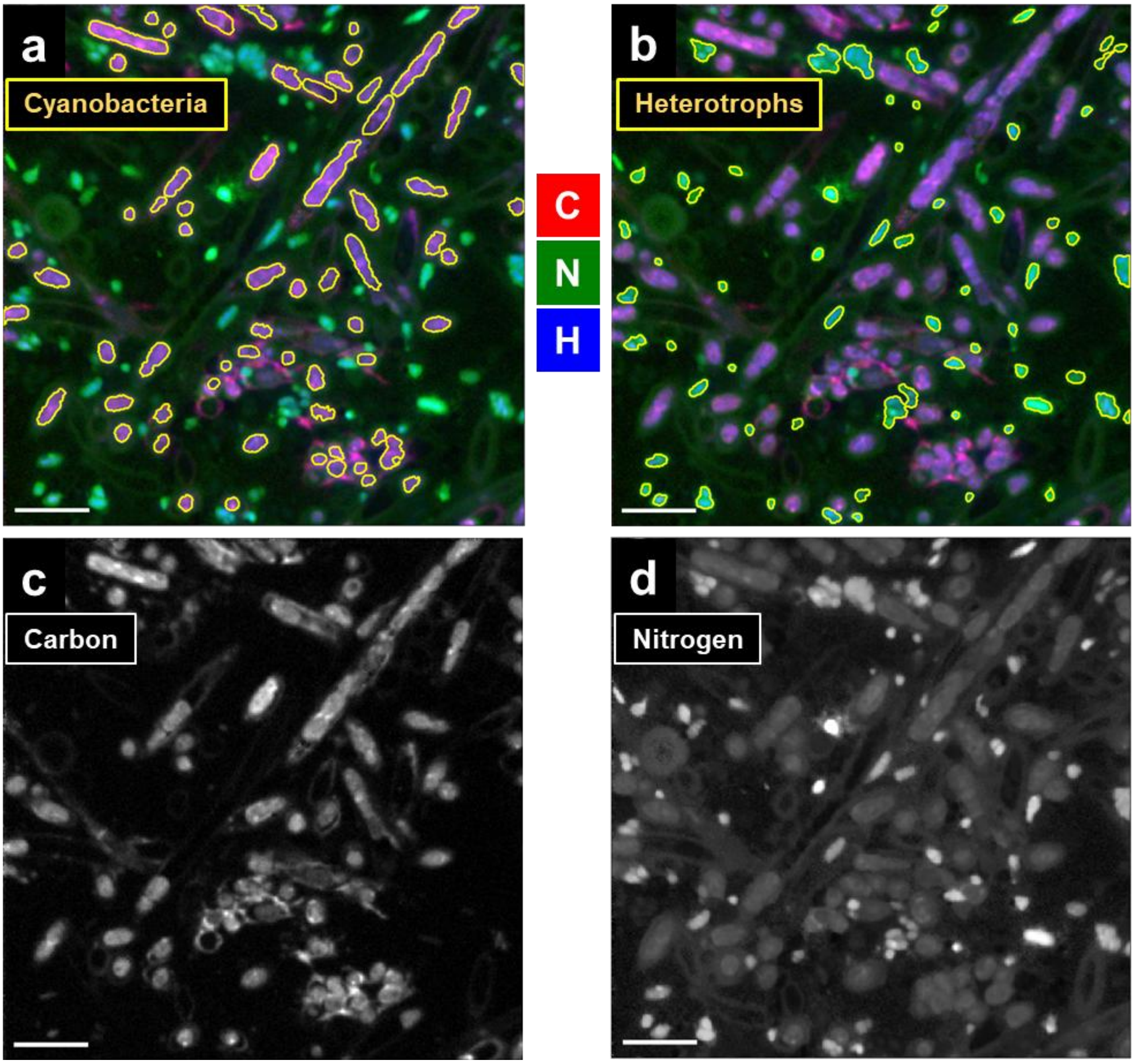

Fig. S7. RGB overlays (a and b): Red – carbon assimilation, Green – nitrogen assimilation, Blue – hydrogen incorporation from heavy water. Cyanobacteria and heterotrophs are defined with yellow RoIs according to their activity in the assimilation of carbon (cyanobacteria) and nitrogen (heterotrophs) shown in frames c and d.

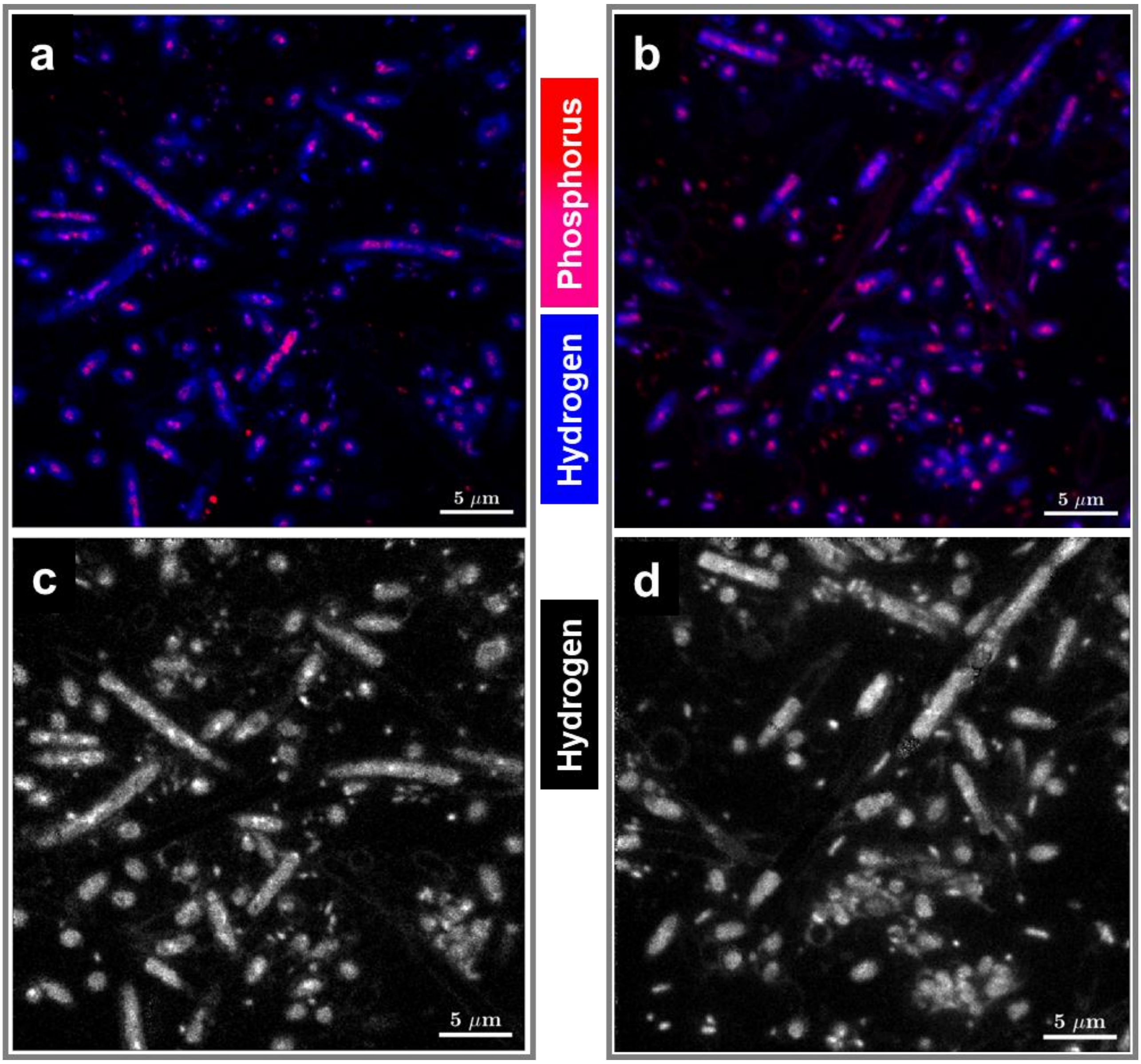

Fig. S8. Hydrogen incorporation maps and their overlays with P proving the H from water to be primarily supplied to the sites of the most intensive metabolism and biosynthesis like DNA/RNA-synthesis sites in and around bacterial chromosome where ^{2}H is to be bound with N and P.

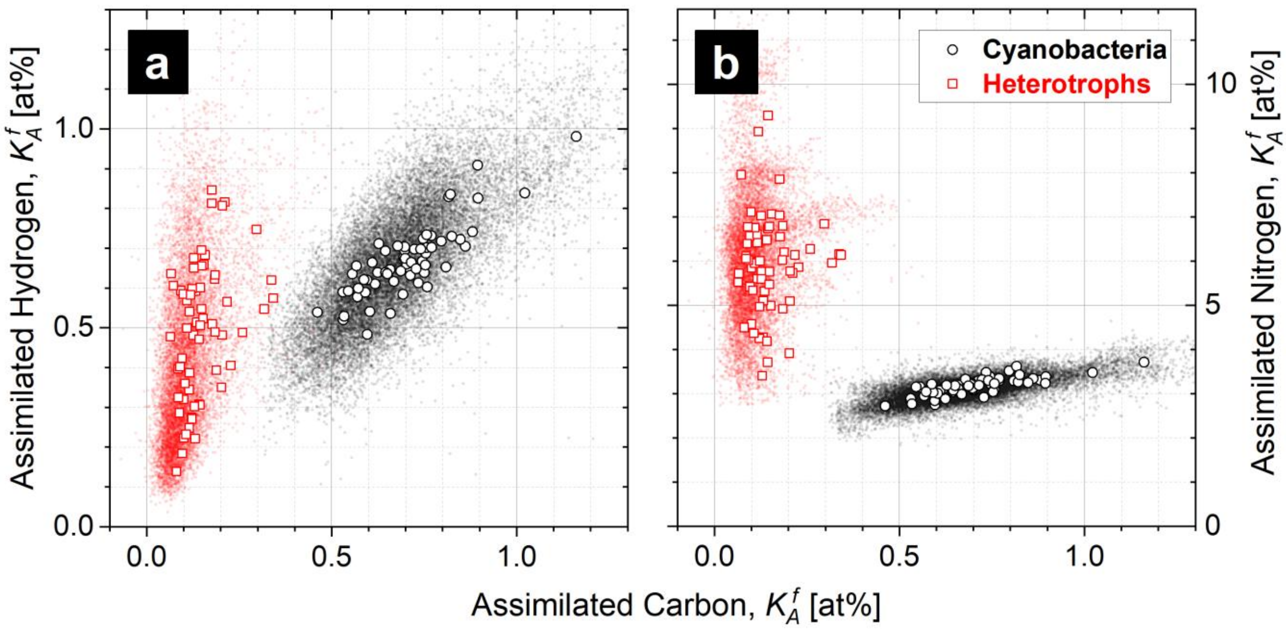

Fig. S9. Relative metabolic activity K_A^f of complex microbial consortium in oxygenic photogranules (OPG) revealed with multi-isotope (^{2}H, ^{13}C, ^{15}N) SIP-nanoSIMS. Scatterplots of pixels assigned to the biomass of autotrophic OPG inhabitants (in black) and to heterotrophs (in red) according to the fraction of assimilated carbon and hydrogen – frame (a); and according to carbon and nitrogen relative assimilation – frame (b). The relative assimilation of single cells is represented with circles for autotrophs and with rectangles for heterotrophs as the mean values over biomass volume-units (voxels) within the corresponding cell-confining RoIs (shown with yellow contours in Figs. S8 a and S8 b).

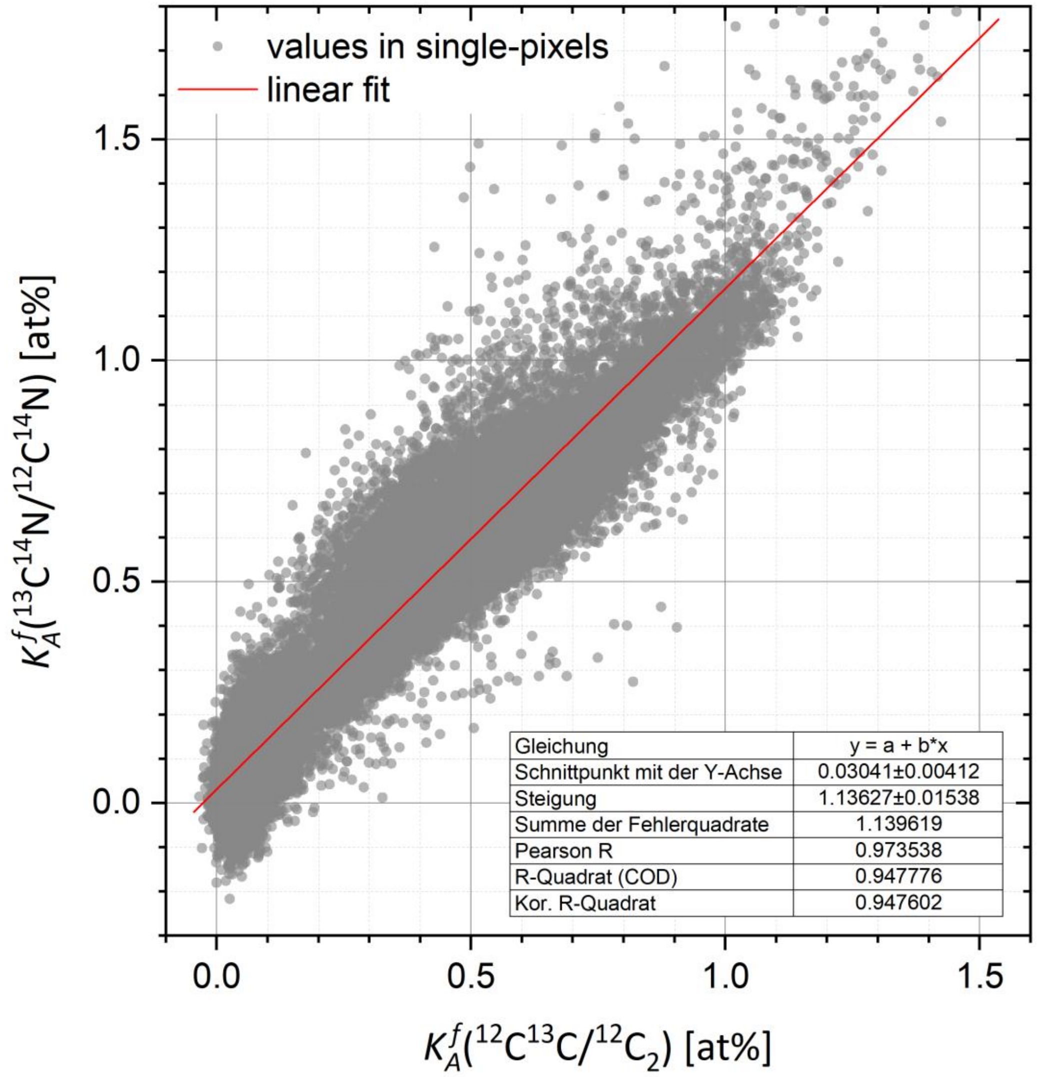

Fig. S10. Linear fit of $K_A^f\,(^{13}\mathrm{C}^{14}\mathrm{N}/^{12}\mathrm{C}^{14}\mathrm{N}) = a + b \times K_A^f\,(^{12}\mathrm{C}^{13}\mathrm{C}/^{12}\mathrm{C}_2)$ relation providing the slope *b* and y-intercept *a* as 1.136 multiplication factor and 0.03 at% offset to correct the values of relative assimilation, computed with corresponding cell-related C_2 and C_2H isotopologue ratios, for the dilution of ^{13}C and ^{2}H isotope-tracers with fixative and embedding materials.

References

Bardin, Noémie, Jean Duprat, Georges Slodzian, Ting-Di Wu, Donia Baklouti, Emmanuel Dartois, Rosario Brunetto, Cécile Engrand, and Jean-Luc Guerquin-Kern. 2015. 'Hydrogen isotopic fractionation in secondary ion mass spectrometry using polyatomic ions', International Journal of Mass Spectrometry, 393: 17-24.

Berry, D., E. Mader, T. K. Lee, D. Woebken, Y. Wang, D. Zhu, M. Palatinszky, A. Schintlmeister, M. C. Schmid, B. T. Hanson, N. Shterzer, I. Mizrahi, I. Rauch, T. Decker, T. Bocklitz, J. Popp, C. M. Gibson, P. W. Fowler, W. E. Huang, and M. Wagner. 2015. 'Tracking heavy water (D2O) incorporation for identifying and sorting active microbial cells', Proc Natl Acad Sci U S A, 112: E194-203.

Guillermier, C., J. C. Poczatek, W. R. Taylor, and M. L. Steinhauser. 2017. 'Quantitative imaging of deuterated metabolic tracers in biological tissues with nanoscale secondary ion mass spectrometry', Int J Mass Spectrom, 422: 42-50.

Kopf, S. H., S. E. McGlynn, A. Green-Saxena, Y. Guan, D. K. Newman, and V. J. Orphan. 2015. 'Heavy water and ^{15}N labelling with NanoSIMS analysis reveals growth rate-dependent metabolic heterogeneity in chemostats', Environ Microbiol, 17: 2542-56.

Polerecky, L., B. Adam, J. Milucka, N. Musat, T. Vagner, and M. M. Kuypers. 2012. 'Look@NanoSIMS--a tool for the analysis of nanoSIMS data in environmental microbiology', Environ Microbiol, 14: 1009-23.
